# All intrinsically active Erk1/2 mutants autophosphorylate threonine207/188, a plausible regulator of the TEY motif phosphorylation

**DOI:** 10.1016/j.jbc.2025.108509

**Published:** 2025-04-11

**Authors:** Alexey Baskin, Nadine Soudah, Nechama Gilad, Neriya Halevi, Ilona Darlyuk-Saadon, Hanan Schoffman, David Engelberg

**Affiliations:** 1 Department of Biological Chemistry, The Institute of Life Science, The Hebrew University of Jerusalem, Jerusalem, Israel; 2 Singapore-HUJ Alliance for Research and Enterprise, Mechanisms of Liver Inflammatory Diseases Program, National University of Singapore, Singapore; 3 Stein Family Mass Spectrometry Unit, The Research Infrastructure Center, The Institute of Life Science, The Hebrew University of Jerusalem, Jerusalem, Israel; 4 Department of Microbiology and Immunology, Yong Loo Lin School of Medicine, National University of Singapore, Singapore

**Keywords:** eukaryotic protein kinases (EPKs), MAP kinases, Erk, activation loop, phosphorylation, TEY motif, myopathy, skeletal muscle atrophy

## Abstract

The extracellular-activated kinases 1 & 2 (Erk1/2) are catalytically active when dually phosphorylated on a TEY motif located at the activation loop. In human patients with cardiac hypertrophy, Erk1/2 are phosphorylated on yet another activation loop's residue, T207/188. Intrinsically active variants of Erk1/2, mutated at R84/65, are also (auto)phosphorylated on T207/188. It is not known whether T207/188 phosphorylation is restricted to these cases, nor how it affects Erks' activity. We report that T207/188 phosphorylation is not rare, as we found that: 1) All known auto-activated Erk1/2 variants are phosphorylated on T207/188. 2) It occurs in various cell lines and mouse tissues. 3) It is extremely high in patients with skeletal muscle atrophies or myopathies. We propose that T207/188 controls the permissiveness of the TEY motif for phosphorylation because T207/188-mutated Erk1/2 and the yeast Erk/Mpk1 were efficiently dually phosphorylated when expressed in HEK293 or yeast cells, respectively. The T207/188-mutated Mpk1 was not TEY-phosphorylated in cells knocked out for MEKs, suggesting that its enhanced phosphorylation in wild-type cells is MEK-dependent. Thus, as T207/188-mutated Erk1/2 and Mpk1 recruit MEKs, the role of T207/188 is to impede MEKs' ability to phosphorylate Erks. T207/188 also impedes autophosphorylation as recombinant Erk2 mutated at T188 is spontaneously autophosphorylated, although exclusively on Y185. The role of T207/188 in regulating activation loop phosphorylation may be common to most Ser/Thr kinases, as 86% of them (in the human kinome) possess T207/188 orthologs, and 160 of them were already reported to be phosphorylated on this residue.

Eukaryotic protein kinases (EPKs) modify properties of numerous proteins *via* reversible phosphorylations and are thereby involved in regulating all physiological processes. EPKs reside in an equilibrium between several conformations, most prominent of which seem to be active and inactive. For most EPKs, the equilibrium is shifted towards the active conformation upon phosphorylation of a particular residue (in Ser/Thr kinases, it is commonly a threonine) within a domain known as the activation loop. This phosphorylation induces an array of new intra-protein interactions that culminate in the assembly of imaginary transverse spines, termed the regulatory and the catalytic spines. The catalytic spine is completed once ATP is bound, stabilizing the active conformation (1, 2, 3). In many EPKs, activation loop phosphorylation can occur *via* a spontaneous intrinsic reaction, autophosphorylation, whose mechanism is not fully understood (4). It is further not clear what is the basis for the more efficient autophosphorylation of some EPKs and the poor autophosphorylation of others.

This study addresses the role of another conserved threonine residue (T197 in PKA, T207/188 in Erk1/2), residing very close to the “activatory” threonine (Fig. 1). This threonine was already noticed by several investigators to be important for catalysis, and to be itself phosphorylated in many EPKs (Table S1). But its exact role and the effect of its phosphorylation have remained unclear. This threonine (will be termed from now on T207/188, on the basis of Erk1/2 numerations) is not required for assembly of the spines, or the active conformation. But, in most EPKs it interacts with a critical Asp, which functions as an activator of substrates' hydroxyls rendering them nucleophilic, capable of attacking ATP. This Asp (D147 in Erk2) is termed, therefore, the catalytic Asp residue (ׁFig. 1). T207/188 also interacts with a Lys residue of the active site (Lys149; Fig. 1), a single link from the C lobe to phosphate of ATP, which is essential for phosphoryl transfer in many Ser/Thr kinases (5, 6). Deciphering the role of T207/188 phosphorylation is far from trivial because of three major reasons. First, although this site was found phosphorylated in many EPKs in various cell types and tissues (Table S1), for no EPK it is known how to induce this phosphorylation. Second, because it is not known how to obtain kinases phosphorylated in this residue, one cannot measure its impact on either structure or activity. Third, studying this phosphoacceptor *via* mutagenesis is futile because mutating this threonine was shown, in a large number of kinases, to impair catalytic capabilities altogether (see Table 1; see also in (7)).

**Figure 1:**
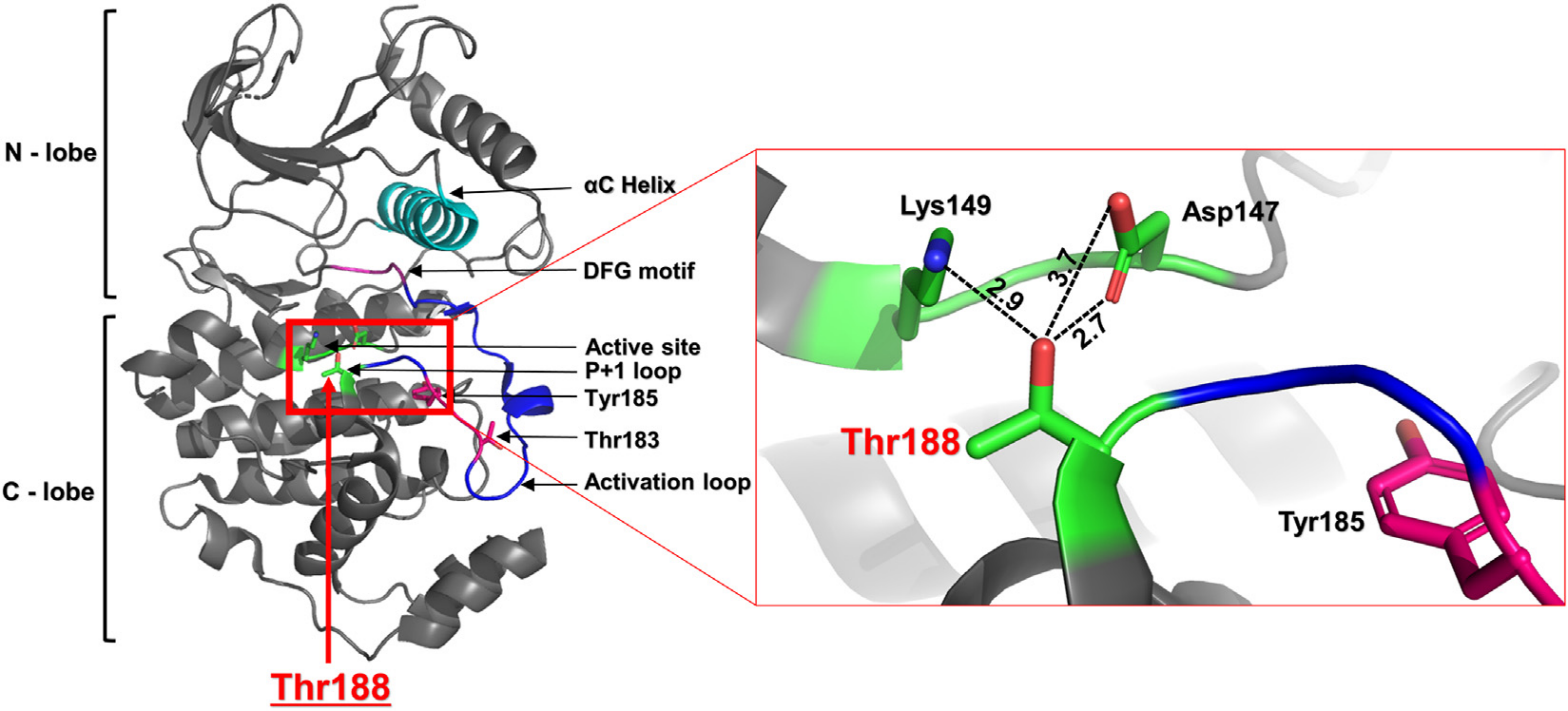
**Thr188 is located in the vicinity of critical residues within the crystal structure of non-phosphorylated Erk2.** The structure of inactive human Erk2 (PDB:4S31) is shown in a ribbon representation mode. Position of significant domains and residues is marked.

**Table 1:** Summary of literature analysis of studies performed on T188-orthologs in Ser/Thr kinases (LOF = loss-of-function)

Kinase	Sub-family within EPKs	Organism	Phosphosite	Effect of mutation	References
ASK1 (MAP3K5)	STE	Human\Murinae	T842	LOF	(52, 53)
AurB	CaMK	Human	T236	LOF	(54, 55)
AurC	CaMK	Human	T202	LOF	(56)
BRSK1	CaMK	*C. elegans*	S193	LOF	(57)
CAMK2B	CaMK	Murinae	T177	LOF	(58)
CAMK4	CaMK	Murinae	T204	LOF	(59)
CDC42BPA (MRSKA)	AGC	Human	T240	LOF	(60)
CDC7	Other	Human	T376	LOF	(61, 62)
Chk2 (CHEK2)	CaMK	Human	T387	LOF	(63, 64)
CK1G2 (CSNK1G2)	CK1	Murinae	T215	LOF	(65)
COT(MAP3K8)	STE	Human	T290	LOF	(66, 67)
DAPK3 (ZIPK)	CaMK	Human	T180	LOF	(68)
ERK1 (MAPK3)	CMGC	Murinae	T207	LOF	(7, 10)
ERK2 (MAPK1)	CMGC	Murinae	T190	LOF	(7, 10)
HGK (MAP4K4)	STE	Human	T191	LOF	(69, 70)
HIPK2	CMGC	Human	S357	LOF	(71)
HPK1 (MAP4K1)	STE	Human	T175	LOF	(72)
HRI (EIF2AK1)	Other	Murinae	T490	LOF	(73)
IRAK1	TKL	Human	T387	LOF	(74, 75, 76)
LRRK1	TKL	Human	T1400	LOF	(77, 78)
LRRK2	TKL	Human	T2035	LOF	(79, 80)
MARK1	CaMK	Murinae	S219	LOF	(81)
MARK2	CaMK	Human	S212	LOF	(82)
MARK3	CaMK	Human	S215	LOF	(83, 84)
MEKK1 (MAP3K1)	STE	Murinae	T572	LOF	(85)
MEKK2 (MAP3K2)	STE	Human	T523	LOF	(86)
MELK	CaMK	Human	S171	LOF	(87)
MINK1	STE	Human	T191	LOF	(88)
MLK1 (MAP3K9)	TKL	Human	T312	LOF	(89)
MST3 (STK24)	STE	Murinae	T182	LOF	(90)
MYO3A	STE	Human	T188	LOF	(91)
NEK3	NEK	Human	T165	LOF	(92)
NEK6	NEK	Murinae	T210	LOF	(93)
NIK (MAP3K14)	STE	Human	T559	LOF	(94)
P38α (MAPK14)	CMGC	Human	T185	a	(35)
PKA	CaMK	Human	T201	LOF	(95)
PKR (EIF2AK2)	Other	Human	T451	LOF	(96)
PLK1	CaMK	Human	T214	LOF	(97)
RIPK3	TKL	Human	T182	LOF	(98)
RIPK4	TKL	Murinae	T184	LOF	(99)
SIK1	CaMK	Murinae	S186	LOF	(100, 101)
SLK	STE	Human	T193	LOF	(102)
STLK3 (STK39)	STE	Human	T236	LOF	(103)
TAK1 (MAP3K7)	TKL	Human	S192	LOF	(104, 105)
TESK1	TKL	Murinae	S215	LOF	(106)
TTK (MPS1)	Other	Human	T686	LOF	(107)

a In p38α the mutation abolished autophosphorylation, but not MKK6-induced activity.

Our impetus to study T207/188 phosphorylation was raised by the observation that in intrinsically active variants of Erk1/2 that we generated, mutated in R84/65 (8, 9, 10), it is spontaneously phosphorylated (10). Further strengthening the need for understanding this phosphorylation are reports that T188 of Erk2 is phosphorylated in the heart of patients with cardiac hypertrophy and some cancer-derived cell lines (8, 11). Finally, as is shown in this study, T188 phosphorylation of Erk2 is not a peculiar event, occurring in a certain disease or in a particular active mutant, but rather occurs in all *bona fide* active variants of Erk1/2 (Fig. 2, *A* and *B*) and in many tissues in mammals (Fig. 3).

**Figure 2:**
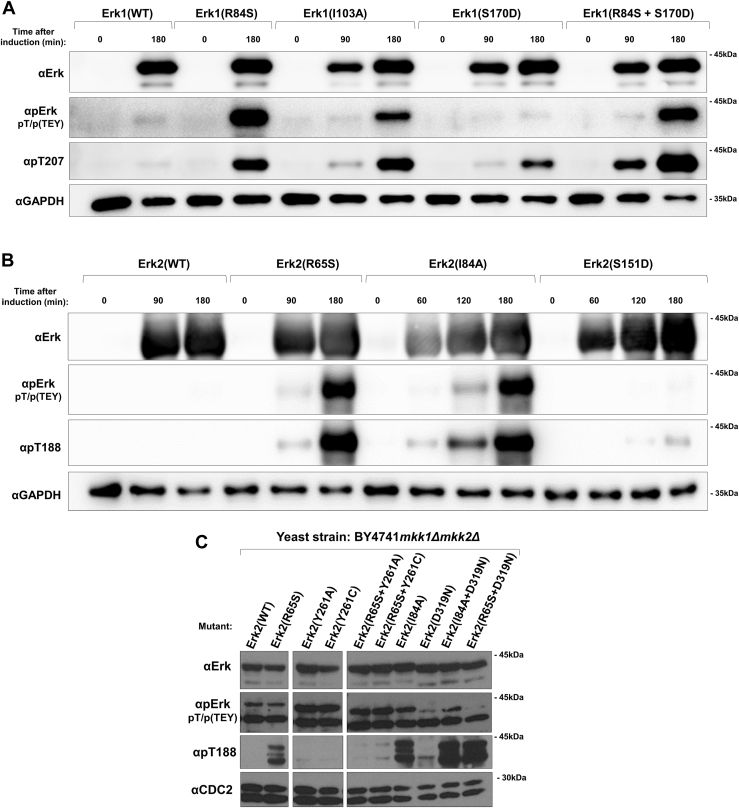
**All currently known intrinsically active mutants of Erk1/2 autophosphorylate T207/188 when expressed in *E. coli* or in the yeast *Saccharomyces cerevisiae*.** The indicated Erk1 (*A*) or Erk2 (*B*) molecules were inducibly expressed in *E. coli* cells. Samples were removed from the cultures at the indicated timepoints post-induction, and cell lysates were prepared and analyzed by Western blot with the indicated antibodies. *C*, the indicated mammalian Erk2 molecules were expressed in the yeast *mkk1Δmkk2Δ* strain, lacking the genes encoding the MEKs Mkk1 and Mkk2. Cultures were grown to logarithmic phase on YNB(–URA), cell lysates were prepared and analyzed by Western blot with the indicated antibodies. Experiments were performed twice with similar results.

**Figure 3:**
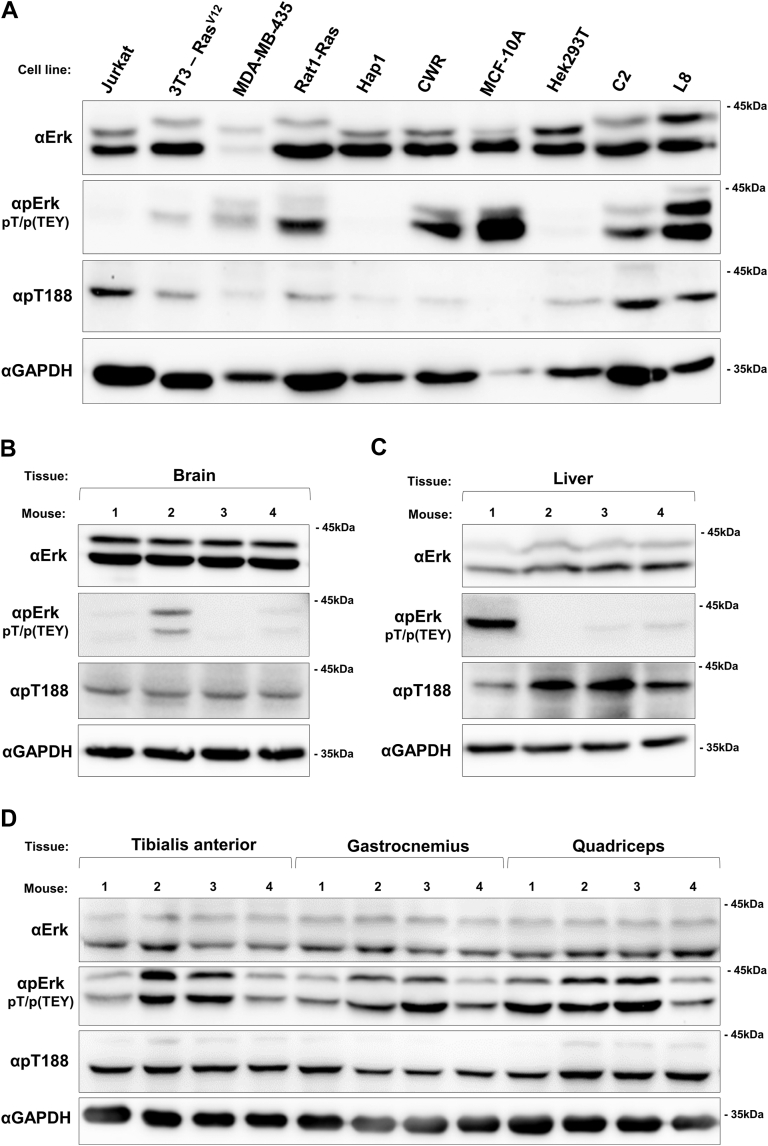
**Low levels of T188-phosphorylated Erk2 are monitored in various cell lines and in mouse tissues.***A*, cell lysates prepared from the indicated cell lines were subjected to Western blot analysis with the indicated antibodies. *B*, *C*, and *D*, Protein lysates were prepared from mice brain (*B*), liver (*C*), tibialis anterior, gastrocnemius, and quadriceps (*D*) (n = 4), and analyzed by Western blot with the indicated antibodies.

Erk1/2 are expressed in all eukaryotic cells, can phosphorylate more than 500 substrates, and are involved in regulating most cellular processes (12). They are activated in response to growth factors *via* the proto-oncogenic RTK-Ras-Raf-MEK cascade and are consequently associated with various Rasopathies and most cancers (13). The only known mode of regulating catalysis of Erk1/2 is phosphorylation and dephosphorylation of the activation loop. Phosphorylation is catalyzed by MAPK kinases, known as MEK1 and MEK2, while dephosphorylation could be achieved *via* various phosphatases (14, 15).

Different from other EPKs, activation loop's phosphorylation in Erk1/2 occurs on two residues, on a sequence known as the TEY motif (T^202/183^EY^204/185^ in Erk1/2) (Fig. 1). T207/188, also residing in the activation loop, is not affected by growth or differentiation factors and it is not a substrate of MEKs (10). T207/188 resides in a region of the activation loop termed the P+1 loop (Fig. 1), which situates the substrate's phosphoacceptor for phosphorylation.

Similar to observations in other EPKs, mutating T207/188 in Erk1/2 to Ala or Glu also abolished catalysis. But, the inactive T207/188-mutated Erk1/2 molecules, tested as purified recombinant proteins, were reported to display an increase in phosphorylation of the TEY motifs (based on Western blot with anti-phos(TEY)Erk antibodies) (7, 8, 12). Here, we characterized this phosphorylation in cells and in purified proteins *via* MS/MS analysis as well as by using site-specific antibodies.

Erks are inefficient autoactivators (16), so that phosphorylation of the TEY motif is absolutely dependent on MEKs (17, 18, 19). Nevertheless, the capability to perform the autophosphorylation/autoactivation reaction does exist in Erks, as is proven by point mutations that de-occlude it (8, 9, 20, 21). For example, almost any mutation in R84/65 renders Erk1/2 capable of autoactivation. These mutations also ignite autophosphorylation on T207/188 (8, 10). Furthermore, several mutants, primarily Erk1^R84S^ and Erk1^R84H^, are oncogenic, capable of transforming cells in culture and of giving rise to tumors in transgenic mice and flies ((8, 10, 22) Soudah 2025 (submitted)). The R84H mutation in ERK1 is also found in cancer patients (23, 24).

Several more active mutants of Erk1/2 have been reported, some proven to be intrinsically active catalytically (20, 21), including Erk1/2^I103/84A^ and Erk1/2^S170/151D^. They were shown to render Erk1/2 autophosphorylatable on the TEY motif and consequently catalytically active (20, 21). The status of T207/188 in all these mutants has not been reported. Other variants were isolated as gain-of-function (GOF) mutants in genetic screens and were not fully characterized [*e.g.*, Brenan *et al.*, 2016 (25); For comprehensive reviews on Erk active mutants, see references (24, 26)].

As mentioned, attempts to study the role of T207/188 by mutating it led to total elimination of catalytic activity (7, 10, 21, 27, 28), but on the other hand, when expressed as a transgene in mice heart, Erk2^T188D^ seems active, rendering the mice prone to cardiac hypertrophy (11). Here we exhausted the mutagenesis approach and replaced T207/188 in Erk1^WT^, Erk1^R84H^, Erk2^R65H^, with Ala, Glu or Asp and in Erk2^WT^, with Ala, Glu, Asp, Ser, Asn, and Cys. Mutants were tested *in vitro* as purified recombinant proteins and in HEK293 cells. Ala, Glu and Asp mutants were also tested for capability to oncogenically transform NIH3T3 cells. *In vitro* kinase assay with recombinant proteins showed that all mutants except Erk2^T188S^ and Erk2^T188C^ lost catalytic activity, although they were spontaneously autophosphorylated. MS/MS analysis revealed that this phosphorylation occurs on tyrosine only explaining the lack of activity. When tested in HEK293 mutants were spontaneously dually phosphorylated to some degree, but T207-mutated Erk1^R84S^ and Erk1^R84H^ lost oncogenic activity. We further mutated the residue equivalent to T207/188 in the yeast MAPK Erk/Mpk1 and observed that the substitutions resulted in a dramatic increase of dual TEY phosphorylation, which was MEK-dependent. It seems that T207/188 is controlling the level of TEY phosphorylation with different effects on MEK-dependent- and auto-phosphorylation. AlphaFold-based structure of T188-phosphorylated Erk2 shows that phosphorylated T188 is shifted from its original position, disrupting its interaction with the catalytic Asp (D147). We propose a model in which T207/188 controls availability of the “activatory” TEY motif for phosphorylation. Perhaps T207/188 phosphorylation liberates the catalytic Asp to activate TEY residues.

Our kinome-wide bioinformatic study shows that T207/188 is conserved in the sequence of all MAPKs in various kingdoms with no exception, and in 86% of the human Ser/Thr kinases. Rigorous analysis of published phosphoproteomic data, combined with a large-scale literature survey, showed that in many Ser/Thr kinases the T207/188 ortholog is phosphorylated and is critical for catalysis, just as was found for Erk1/2. We speculate that perhaps in many (all) of these kinases, phosphorylation of T207/188 orthologs controls phosphorylation of the activatory phosphoacceptor.

## Results

### All currently known intrinsically active Erk1/2 mutants autophosphorylate T207/188

Erk1/2 were reported to be phosphorylated on T207/188 in human patients and mouse models of cardiac hypertrophy and on intrinsically active Erk1/2 molecules mutated at R84/65 (10, 11). To assess whether this phosphorylation is peculiar, unique to these cases, or is rather more general, we first tested the other known catalytically intrinsically active Erks, *i.e.*, Erk1^I103A^, Erk1^S170D^, Erk2^I84A^ and Erk2^S151D^ (10, 20, 21). A Western blot assay was performed on lysates prepared from *E. coli* cells expressing these Erk mutants. While Erk1^WT^ and Erk2^WT^ were not phosphorylated on either the TEY motif or T207/188, Erk1^I103A^, Erk1^S170D^, Erk2^I84A^ and Erk2^S151D^ were phosphorylated on both the TEY motif and the T207/188 residue concomitantly with their expression, similarly to Erk1^R84S^ and Erk2^R65S^ that served as positive controls (Fig. 2, *A* and *B*). Notably, the intrinsic activity of Erk1^S170D^ and Erk2^S151D^ is weak as their capability of autophosphorylating the TEY motif is low. Yet, they efficiently autophosphorylate T207/188 (Fig. 2, *A* and *B*). We further expressed Erk2^I84A^ and Erk2^R65S^ in cells of the yeast *Saccharomyces cerevisiae,* knocked out for *MKK1* and *MKK2* encoding the yeast MEKs. Erk2^I84A^ and Erk2^R65S^ were found to be phosphorylated on T188 in these yeast cells (Fig. 2*C*). This phosphorylation is clearly a consequence of autophosphorylation, as combining the R65S mutation with mutations that impede catalytic activity (mutations in Y261) prevents T188 phosphorylation (Fig. 2*C*). Thus, T207/188 phosphorylation is not specific to Erk1/2 molecules mutated on R84/65 but seems to be a property common to all catalytically intrinsically active, self-activating Erk1/2 mutants. The observations that the intrinsically active Erk mutants are phosphorylated on T207/188 as recombinant proteins expressed in *E. coli* or in *S. cerevisiae* cells knocked out for the relevant MEKs suggest that phosphorylation of this residue is a result of intrinsic activity.

### Other reported gain-of-function mutants of Erk1/2 are not intrinsically active catalytically and are not phosphorylated on T207/188

As we found that all currently known Erk1/2 mutants that were proven to be *bona fide* intrinsically catalytically active autophosphorylate T207/188, we checked whether other variants of Erks, reported to be active, may also autophosphorylate this residue. We thus inserted into Erk1 and Erk2 the mutations reported to bestow gain-of-function properties on these kinases, *i.e.*, P75G, P75L, E98K, S159L, G186D (in Erk1), P55G, P55L, E79K, S140L and G167D (in Erk2) (25, 29). The activity and phosphorylation status of the mutants were tested on purified recombinant proteins and on proteins expressed in HEK293 cells. We also tested their ability to oncogenically transform NIH3T3 cells. The results, described in detail in “Supporting information,” show that the mutants did not acquire any unusual intrinsic spontaneous activity and behaved just like Erk1/2^WT^ (see Supplementary Results and Figs. S1–S3). Thus, T207/188 phosphorylation is associated with molecules that acquired *bona fide* intrinsic autophosphorylation capability and could be considered a marker for intrinsically active Erks. It does not occur in the proposed GOF mutants that probably function *via* a mechanism that does not involve changes in catalytic properties.

### Phosphorylation of T188 of Erk2 is monitored in various cell lines, mouse tissues, and skeletal muscle of human patients with inflammatory diseases

Having observed that T188 phosphorylation of Erk2 is more widespread than presumed, occurring in all catalytically active mutants, we checked for this phosphorylation in native Erk1/2, in living cells in culture, and in mouse tissues. In all cell lines tested, phosphorylation of T188 was detected, although at very low levels. Some higher levels were monitored in the myogenic cell lines L8 and C2C12 (Fig. 3*A*). In mouse tissues we measured low levels of T188 phosphorylation in brain (Fig. 3*B*) and liver (Fig. 3*C*), and higher levels in three different skeletal muscles (Fig. 3*D*). Notably, T207-phosphorylated Erk1 was usually not detected, except for low levels in tibialis anterior and quadriceps (Fig. 3*D*). We cannot conclude whether Erk1 molecules are not phosphorylated on T207 or that steady-state levels of Erk1 are very low so that the fraction phosphorylated on T207 cannot be detected.

**Figure 4:**
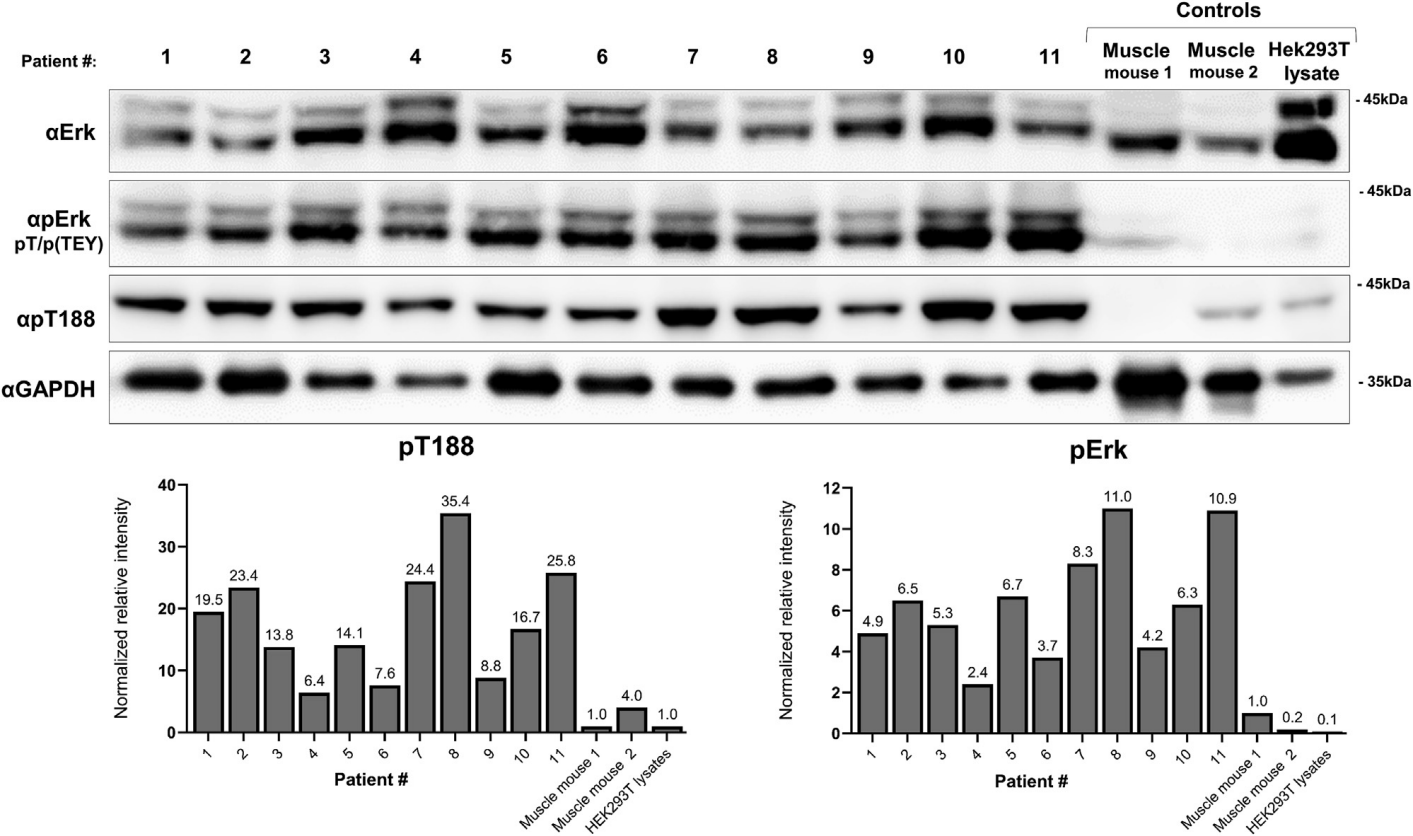
**High levels of T188-phosphorylated Erk2 are monitored in skeletal muscle of human patients suffering of muscle myopathy or atrophy.** Protein lysates were prepared from biopsies removed form skeletal muscle (quadriceps, biceps or deltoid) of 11 patients suffering of the following diseases: 1: Inflammatory myopathy, female, age 50; 2: Immune mediated necrotizing myopathy, male, age 64; 3: Neurogenic atrophy, male, age 59: 4: Inclusion body myositis, male, age 83; 5: Neurogenic muscle atrophy with secondary myopathy, female, age 72; 6: Inflammatory myopathy, male, age 77; 7: Immune mediated necrotizing myopathy, female, age 71; 8: Inflammatory myopathy, male, age 57; 9: Immune mediated necrotizing myopathy associated with type 2 myofibril atrophy, female, age 63; 10: Inflammatory myopathy with neurogenic myopathy, male, age 67; 11: Necrotizing myopathy with DD, male, age 58 and indicated experimental controls. Lysates were analyzed by Western blot with the indicated antibodies. Lysates prepared from mouse Tibialis anterior muscle and HEK293 cells were analyzed in parallel for comparison. Graphs show quantitation of levels of pErk(TEY) and pT188 relative to levels of mouse muscle that was taken as 1.

As levels of T188 phosphorylation are somewhat higher in myogenic cell lines and in mouse skeletal muscle, we tested muscle tissues of human patients. We were able to obtain 11 muscle biopsies removed from patients with myopathies or atrophies, and in all of them, we monitored very high levels of T188-phosphorylated Erk2 molecules (6–35-fold higher than the level monitored in mouse muscle (Fig. 4)).

In summary, T188 phosphorylation of Erk2 is not particular to hypertrophic hearts or catalytically active mutants, but occurs in various cell lines and tissues.

### Mutating T207 in Erk1^R84H^ abolishes the protein's capability to oncgonecially transform NIH3T3 cells

The observations above strengthen the importance of understanding the effect of T207/188 phosphorylation. In this regard we first expanded the mutagenesis approach. As mentioned, previous studies showed, using *in vitro* kinase assays with recombinant proteins, that mutating T188 of Erk2 to Ala or Asp, or T207 of Erk1 to Ala or Glu, dramatically reduced activity toward substrates, although causing an increase in autophosphorylation (7, 10, 21, 27, 30). On the other hand, when expressed as a transgene in mice, Erk2^T188D^ enhances cardiac hypertrophy (11), raising the possibility that Erk1/2 proteins mutated in T207/188 are perhaps active *in vivo* although not active as recombinant proteins. We therefore generated a large panel of Erk1/2 molecules, mutated on T207/188 (to Ala, Asp, or Glu) on the background of Erk1/2^WT^, Erk1/2^R65/84S^ or Erk1/2^R65/84H^.

**Figure 5:**
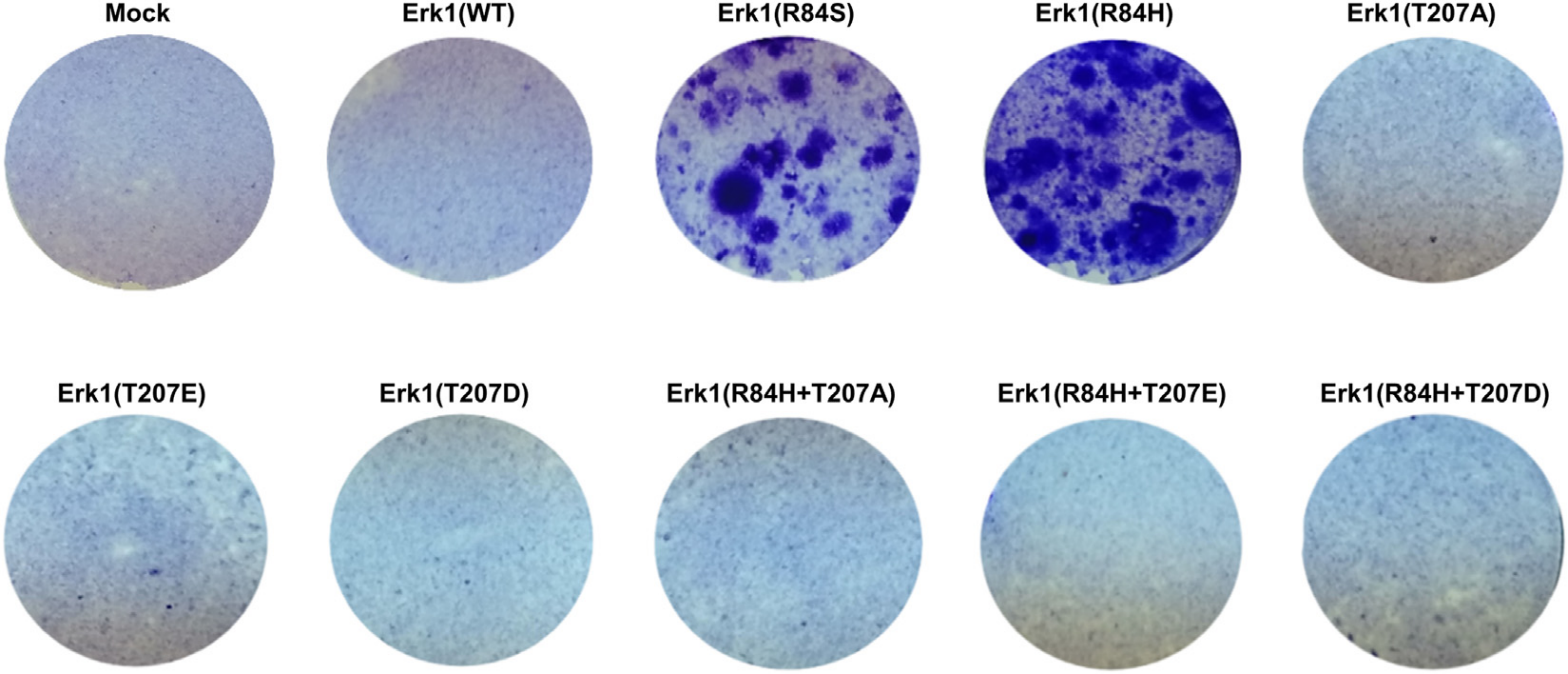
**Mutating T207 in the oncogenic molecules Erk1^R84H^ abolished their ability to oncogenically transform NIH3T3 cells**. NIH3T3 cells were transfected with pCEFL vectors carrying cDNAs encoding the indicated Erk molecules and selection of cells harboring the vector was achieved by addition of G418. Cells were fixed and stained with *crystal violet* 4 weeks after transfection.

We first tested whether mutating T207 in Erk1^R84H^ would affect its pathological capabilities. When expressed in NIH3T3 cells, Erk1^R84H^ gave rise to *foci*, as expected (8), but expression of Erk1^R84H+T207A^, Erk1^R84H+T207E^, or Erk1^R84H+T207D^ did not (Fig. 5). Thus, mutating T207 abolishes the oncogenicity of Erk1^R84H^.

It is concluded that the T207/188 residue is critical for oncogenic activity in living cells. Activity is abolished whether T207/188 is mutated to phosphomimetic or non-phosphorylatable amino acids.

### Mutating T207/188 in Erk1/2 results in elevation of MEK-dependent and independent TEY phosphorylation

**Figure 6:**
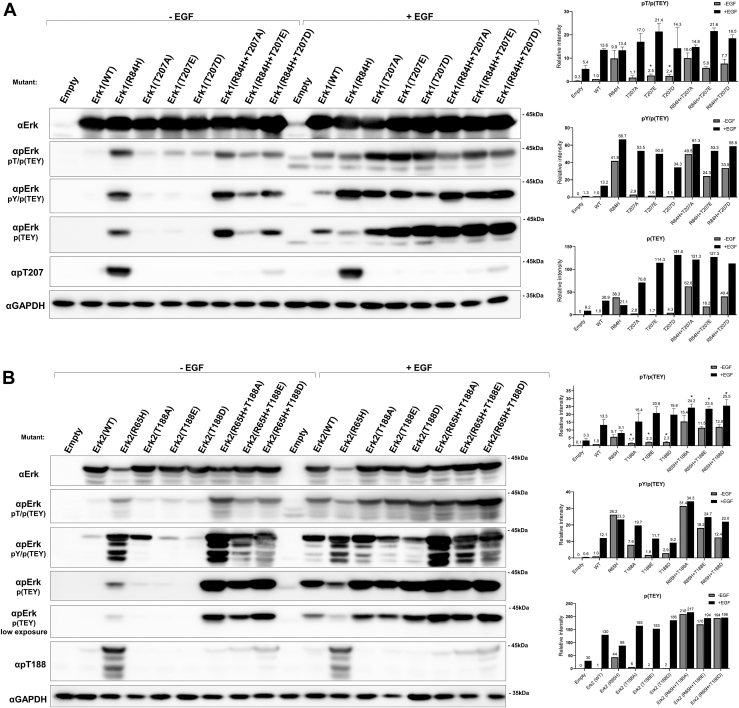
**Mutating T207/188 in Erk1/2 leads to enhanced MEK-dependent and independent TEY phosphorylation in HEK293 cells.** HEK293T cells were transfected with pCEFL vectors carrying the indicated Erk1 (*A*) or Erk2 (*B*) molecules. 48 h post-transfection cells were serum-starved for 16 h and then provided (+) or not provided (−) with EGF (10 min, 50 ng/ml). Cell lysates were prepared and analyzed by Western blot using the indicated antibodies. Erk1/2 phosphorylation levels, as reflected by reacting with each antibody, were quantitated separately using ImageJ densitometry and normalized to levels of Erk1/2^WT^(-EGF) that were set as 1. pY/p(TEY) (lane 3) and p(TEY) (lane 4) experiments were performed twice with similar results. pT/p(TEY) experiment was done more than 3 times. Asterisks mark statistical significance, which was calculated using an unpaired multiple *t* test with *p* < 0.005.

Previous studies noted that mutating T207/188 rendered Erk1/2 capable of autophosphorylating the TEY motif. Most observations were made on recombinant purified proteins and were based on either radioactive kinase assay (10, 21) or the use of anti-phos(TEY)-Erk antibodies. The antibodies used also interact with mono-phosphorylated Erks (7, 21), leaving open questions such as whether the two phosphoacceptors are auto-phosphorylated to the same level, and whether mutating T207/188 affects TEY phosphorylation by MEK. To address these questions, particularly in living cells, we introduced the Erk1/2 mutants to HEK293 cells, which were exposed or not exposed to EGF (for 10 min) 48 h after transfection. Lysates prepared from these cells were reacted, in a Western blot assay, with three different anti-phos(TEY)-Erk antibodies, each recognizing a different phosphorylation state of the TEY motif (31). Antibody #4370 (Cell Signaling) is specific to dually phosphorylated Erks, but also reacts with Erks that are mono-phosphorylated on T202/183 (Fig. 6, row 2). Antibody #4377 (Cell Signaling) reacts with dually phosphorylated Erks and with molecules monophosphorylated on Y204/185 (Fig. 6, row 3). Antibody #M8159 (Sigma-Aldrich) reacts only with dually phosphorylated molecules (Fig. 6, row 4). Specificity of these antibodies was confirmed by reacting them with lysates of HEK293 cells expressing either Erk2^WT(TEY)^, Erk2^AEY^, Erk2^TEF^, or Erk2^AEF^ (Fig. S4).

In cells not exposed to EGF, Erk1 and Erk2 molecules mutated in T207/188 were phosphorylated at somewhat higher level than Erk1^WT^ and Erk2^WT^, (2–6 fold), probably on both the Thr and Tyr of the TEY motif (Figs 6, *A* and *B* see quantitation on the right). Yet, there may be monophosphorylated molecules within the mutants' populations. Erk2^T188A^, for example, seem to contain molecules monophosphorylated on Y185 (Fig. 6*B* 3rd row). The effect of mutating T207/188 on TEY phosphorylation was more prominent in the background of the active variants. Erk1^R84H+T207A^ (Fig. 6*A*) and Erk2^R65H+T188A^ (Fig. 6*B*) molecules are phosphorylated at higher levels than Erk1^R84H^ and Erk2^R65H^. Dual phosphorylation was maintained, but not elevated, in Erk1^R84H+T207D^ and Erk2^R65H+T188D^ and was weaker in Erk1^R84H+T207E^ and Erk2^R65H+T188E^ (Fig. 6, *A* and *B*).

In response to EGF, Erk1^T207A^, Erk1^T207E^, Erk1^T207D^, Erk2^T188A^, Erk2^T188E^ and Erk2^T188D^ were phosphorylated at higher levels than Erk1^WT^ and Erk2^WT^ (Fig. 6). Similarly, Erk1^R84H+T207A^, Erk1^R84H+T207E^ and Erk1^R84H+T207D^ were phosphorylated at higher levels than Erk1^R84H^, and Erk2^R65H+T188A^, Erk2^R65H+T188E^ and Erk2^R65H+T188D^ were phosphorylated at higher levels than Erk2^R65H^. This is manifested best with the anti p(TEY)Erk antibodies (Antibody #M8159) (fourth row in Fig. 6, *A* and *B*). The effect is more pronounced in Erk2 than in Erk1.

Thus, mutations in T207/188 in the background of Erk1^WT^ and Erk2^WT^ ignite a low level of autophosphorylation of the TEY motif when the proteins are expressed in HEK293 cells. A more pronounced effect of the mutations is increasing the efficiency of TEY phosphorylation in response to EGF (probably by MEKs), which is observed in both the backgrounds of Erk1/2^WT^ and Erk1/2^R84/65H^.

T207/188 may thus be a negative regulator of TEY phosphorylation in mammalian Erk1/2. Eliminating T207/188 enhances autophosphorylation to some extent but primarily allows efficient MEK-mediated phosphorylation.

### Mutating the T207/188's equivalent in the yeast Erk/Mpk1 also causes a dramatic increase in TEY phosphorylation

**Figure 7:**
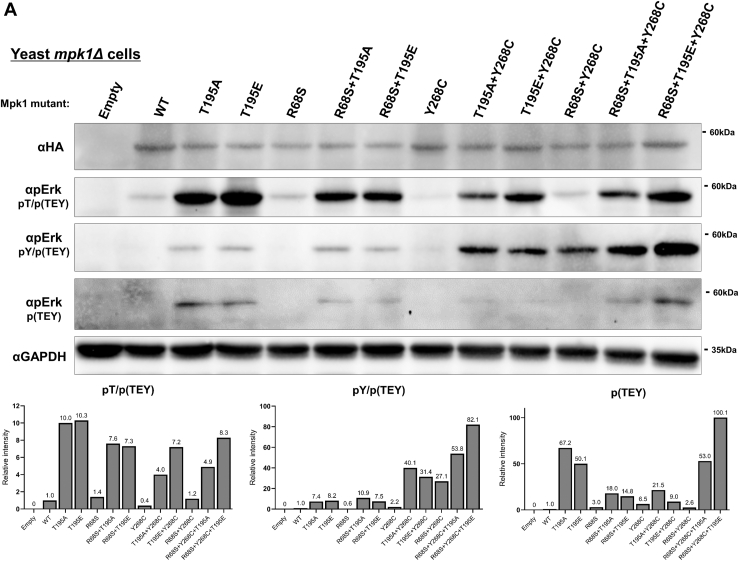

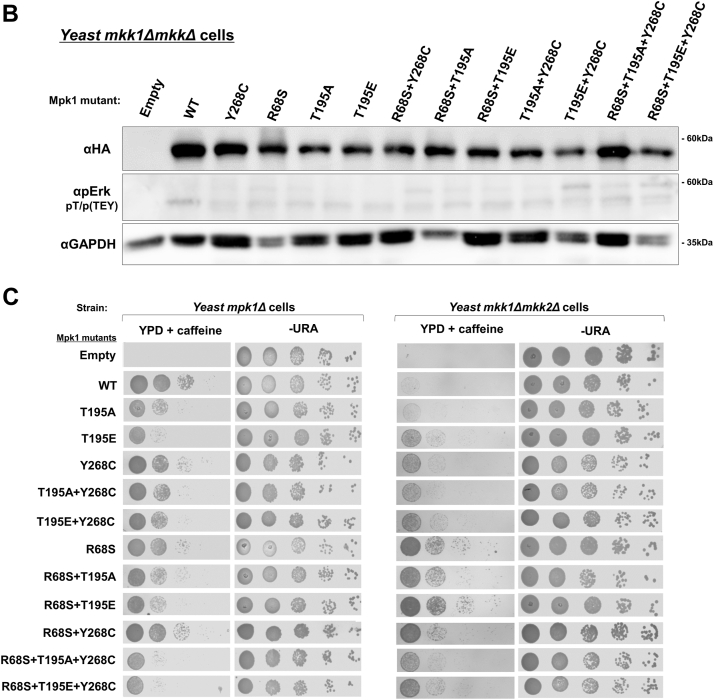
**Mutating the T207/188 equivalent in yeast Mpk1 (T195) leads to a substantial increase in TEY phosphorylation. Biological activity of T195-mutated Mpk1 is not abolished.** AES426 vectors containing the genes encoding the indicated Mpk1 molecules were introduced to yeast *mpk1Δ* (*A*) or *mkk1Δmkk2Δ* (*B*) cells. Cultures were grown to logarithmic phase on YNB(–URA), cell lysates were prepared and subjected to a Western blot analysis with the indicated antibodies. *C*, *mpk1Δ* (*left*) or *mkk1Δmkk2Δ* (*right*) cells expressing the indicated Mpk1 molecules were grown to logarithmic phase on YNB(–URA) and plated in serial decimal dilutions, starting with ∼100,000 cells, on plates containing YPD supplemented with 15 mM caffeine or on plates not containing caffeine (YNB(–URA)). The graphs display the densitometric quantification (ImageJ) of indicated phosphorylation, normalized to the levels of Mpk1^WT^, which were set as 1. The experiment was conducted twice, yielding similar results.

To further study the role of T207/188, we took advantage of the yeast Erk/Mpk1 pathway. Mpk1 is essential for the cell wall integrity (32, 33) and yeast cells lacking the gene encoding Mpk1 (*mpk1Δ* cells) or lacking the gene encoding the relevant MAPK kinases Mkk1 and Mkk2 (*mkk1Δmkk2Δ* cells) cannot proliferate on media supplemented with drugs affecting the cell wall (*e.g.*, caffeine) (31, 32). Intrinsically active variants of Mpk1, such as Mpk1^R68S^ and Mpk1^Y268C^, are active independently of Mkk1/2 and allow *mkk1Δmkk2Δ* cells to proliferate on caffeine (9, 10, 30). We mutated T195, the equivalent site of T207/188, in Erk/Mpk1 and in the intrinsically active variants Mpk1^R68S^, Mpk1^Y268C,^ and Mpk1^R68S+Y268C^. When expressed in *mpk1Δ* cells, all molecules mutated on T195 were phosphorylated at the TEY motif at significantly higher levels than native Mpk1 molecules (Fig. 7*A*). This suggests that in the yeast Erk/Mpk1 too, T195 (its T207/188 ortholog) is a suppressor of TEY phosphorylation. When the Mpk1 mutants were expressed in a strain lacking the relevant MEKs (*mkk1Δmkk2Δ* cells), levels of TEY phosphorylation were not higher in molecules mutated in T195 (Fig. 7*B*), suggesting that mutating T195 enhanced TEY phosphorylation by Mkk1/2 and not *via* autophosphorylation.

Testing *mpk1Δ* cells expressing the Mpk1 mutants on medium supplemented with caffeine showed that the mutation in T195 reduced, but did not abolish, the ability of the Mpk1 molecules to render the cells resistant to caffeine (Fig. 7*C*, left panel, rows 1–3). When expressed in *mkk1Δmkk2Δ* cells, it was observed that substitution of T195 did not interfere with the ability of the intrinsically active variants to support proliferation in the presence of caffeine. Intriguingly, Mpk1^T195E^ manifested properties of an intrinsically active variant (allowed growth of *mkk1Δmkk2Δ* cells in the presence of caffeine; Figure 7*C*, right panel, row 3). Thus, unlike the situation in mammalian Erk1/2, mutating the T207/188 ortholog in the yeast Erk/Mpk1 did not abolish its biological activity.

### MS/MS analysis of Erk2 proteins mutated on T188 revealed spontaneous monophosphorylation of the TEY motif and reduced levels of MEK-mediated phosphorylation

**Figure 8:**
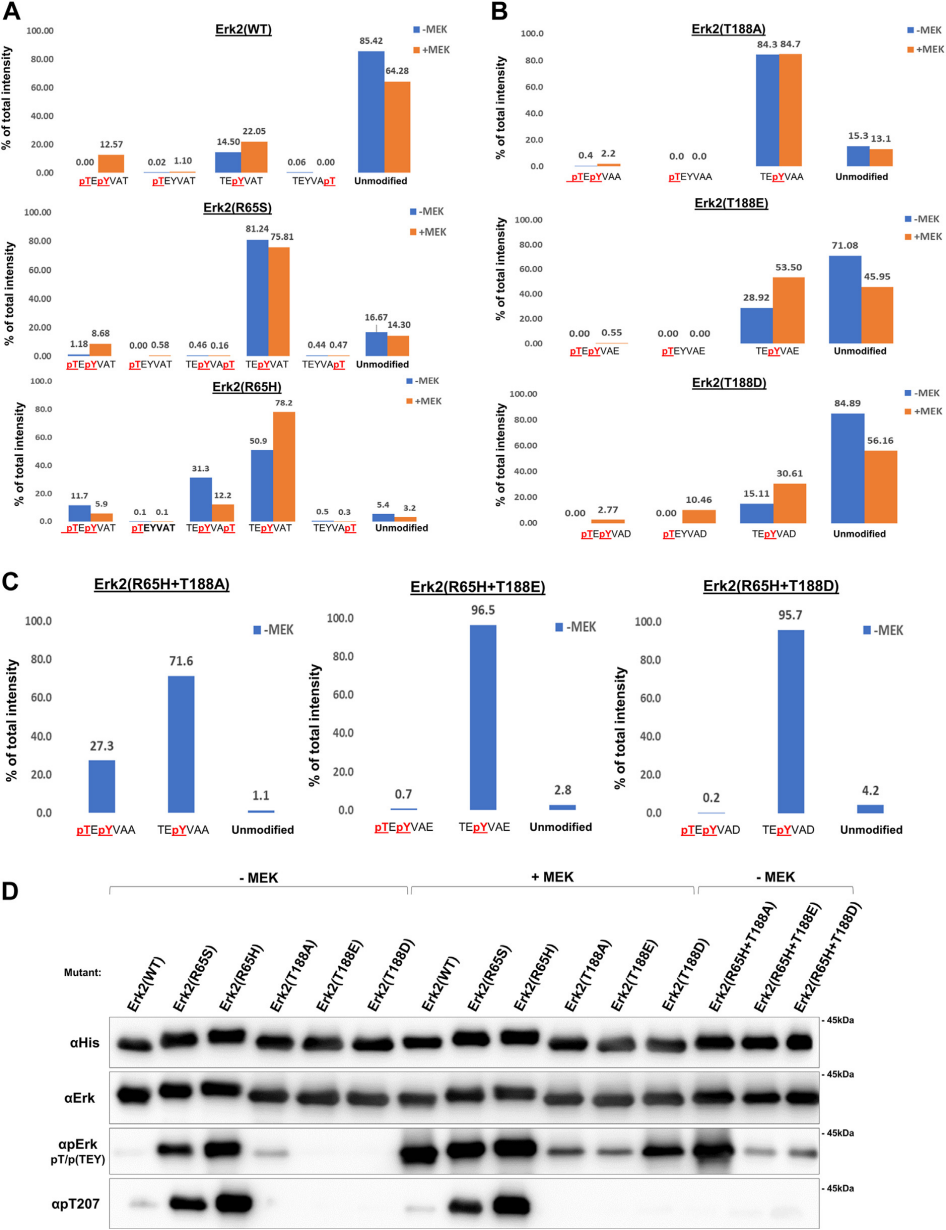
**Purified recombinant Erk2 molecules mutated in T188, are autophosphorylated on Tyr185, and are not efficiently phosphorylated by Mek1^EE^*in vitro*.** The indicated Erk2 molecules, expressed in and purified to homogeneity from *E. coli* cells were subjected or not subjected to phosphorylation by MEK1^EE^ and their phosphorylation status was evaluated *via* Nano LC-MS/MS analysis. *A*, results of MS/MS analysis of Erk2^WT^, Erk2^R65S^ and Erk2^R65H^. *B*, results of MS/MS analysis of Erk2^T188A^, Erk2^T188D^ and Erk2^T188E^. *C*, results of MS/MS analysis of Erk2^R65H+T188A^, Erk2^R65H+T188D^ and Erk2^R65H+T188E^. *D*, Western blot analysis, with the indicated antibodies, performed on the the same molecules tested *via* MS/MS.

The panel of Erk1/2 molecules mutated on T207/188 (to Ala, Asp, or Glu) was also tested, as recombinant purified proteins, in *in vitro* kinase assay. It was observed that all mutants lost catalytic activity whatsoever, either spontaneous (of the active variants) or MEK-dependent (Fig. S5). This observation confirms and complements previous observations that showed lack of catalytic activity of similar mutants (7, 21, 24, 27, 28). As these previous studies also reported spontaneous elevation of TEY phosphorylation in recombinant Erk molecules mutated in 207/188 we applied targeted tandem mass-spectrometry to reveal the exact pattern of this phosphorylation. Recombinant Erk2^WT^, Erk2^R65S^, Erk2^R65H^, Erk2^T188A^, Erk2^T188E^ and Erk2^T188D^ were analyzed as are, or following incubation with active MEK1 (MEK1^EE^; see Experimental procedures). As expected, MS/MS analysis revealed that prior to incubation with MEK^EE^, most of the molecules within the population of Erk2^WT^ (85%) were not phosphorylated. After incubation 12.5% of them were dually phosphorylated and 22% were monophosphorylated on Tyr185 (Fig. 8*A*). Within the populations of the active variants Erk2^R65S^ and Erk2^R65H^ 1.2 and 11.7% of the molecules were spontaneously dually phosphorylated, respectively (Fig. 8*A*). Molecules in these populations were also phosphorylated on T188 alone or on Y185+T188, but, as expected (8), none were phosphorylated on T183+T188 and no triply phosphorylated molecules were identified (Fig. 8*A*).

In the population of Erk2^T188A^ molecules, 84% were monophosphorylated on Tyr185 with or without exposure to MEK1^EE^ (Fig. 8*B*). A small fraction, 2.2%, was doubly phosphorylated by MEK^EE^ and none was monophosphorylated exclusively on Thr183. Erk2^T188E^ was also mostly phosphorylated on Tyr185, but a large fraction of the population was not phosphorylated at all (Fig. 8*B*). The phosphorylation pattern of Erk2^T188D^ was very similar to that of Erk2^WT^ except that it was less efficiently phosphorylated by MEK1^EE^, which dually phosphorylated only 2.2% of the molecules (Fig. 8*B*). On the other hand, MEK1^EE^ was able to monophosphorylate 10.5% of the molecules on Thr183, making Erk2^T188D^ the only Erk molecule tested to be monophosphorylated on Thr183. In summary, MEK1^EE^ was not efficiently phosphorylating all three mutants. This was further confirmed by Western blot analysis performed on the same samples analyzed by MS/MS (Fig. 8*D*, row 3). This observation contrasts with those made in living cells of mammals and yeast, where the substitution of T188 enhanced the efficiency of phosphorylation by MEKs.

We further performed MS/MS analysis on Erk2^R65H+T188A^, Erk2^R65H+T188E,^ and Erk2^R65H+T188D^ proteins. This analysis revealed a significant difference between the effect of the T188A mutation and the T188E and T188D mutations (Fig. 8*C*). Erk2^R65H+T188A^ was efficient in dually autophosphorylating the TEY motif, while Erk2^R65H+T188E^ and Erk2^R65H+T188D^ were not (Fig. 8*C*). Similar results were observed in a Western blot analysis, supporting the MS/MS findings (Fig. 8*D*, row 3).

In summary, mutations in Erk2's and Erk2^R65S^'s T188 also affect TEY phosphorylation when tested as recombinant proteins. But the effect differs in some aspects from that observed in living cells. First, although in both experimental systems mutations in T188 resulted in elevated TEY phosphorylation, in HEK293 and in yeast the molecules were dually phosphorylated, while *in vitro* the strong effect was on Tyr185. Second, while in living cells the mutations in T188 rendered Erk1/2/Mpk1 more permissive for MEK phosphorylation, mutated recombinant Erk2 proteins were less efficiently phosphorylated by MEK^EE^ than Erk2^WT^.

It is thus concluded that mutating T207/188 affects phosphorylation of the TEY motif, but in different manners on purified proteins and on proteins expressed in living cells (see Discussion).

### AlphaFold-based structure of T188-phosphorylated Erk2 suggests re-orientation of phospho-T188, disrupting the interaction with catalytic Asp147

**Figure 9:**
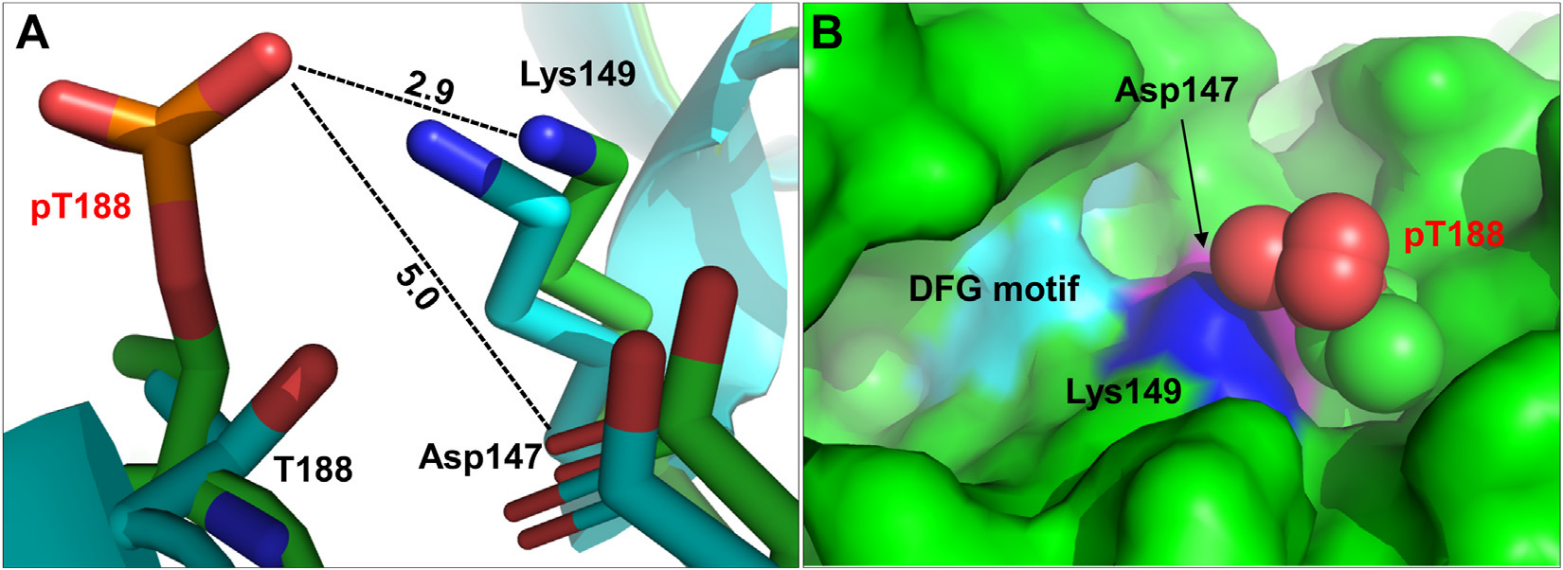
**AlphaFold 3 simulation of T188-phosphorylated Erk2 structure suggests re-orientation of phospho-T188, releasing catalytic Asp147, while maintaining interaction with Lys149.** 3D structure of Erk2^WT^ was generated using AlphaFold 3 and its similarity to available apo crystal structures was confirmed. On the basis of same structure T188-phosphorylated Erk2 was generated. *A*, superimposition of inactive Erk2^WT^ structure (PDB:4S31, *teal*) and AlphaFold 3-generated structure of T188 phosphorylated Erk2 (*green*). Re-orientation of T188 following its phosphorylation, releasing catalytic aspartic while maintaining bonding with Lys149. *B*, surface representation of generated structures with indicated motifs suggests possible obstruction of P+1 motif by phosphorylated T188, possibly interfering with substrate binding or interaction with catalytic residues (Asp147, Lys149, DFG motif).

What could be the structural basis for the regulation of TEY phosphorylation by T188? Until crystal structures of Erks phosphorylated on T207/188 are available AlphaFold 3 simulation may provide some insights. AlphaFold 3-based structure (34) of T188-phosphorylated-Erk2 suggests that phosphorylated T188 is repulsed away from the active site, releasing catalytic Asp but maintaining interaction with Lys149 (Fig. 9) (see Discussion).

### 86% of the Ser/Thr kinases in the human kinome possess a Thr or a Ser, orthologous to Erk1/2's T207/188

T207/188 was already reported, primarily by Lai and Pelech (7) to be a conserved residue, found in many EPKs (see Fig. 6 in reference (7)). This may suggest that its regulatory role in Erk1/2, as proposed here, and its essentiality for catalysis are shared by other EPKs. As a large body of new experimental data has been gathered since the study of Lai and Pelech (7) we re-visited the matter. We determined the degree of T207/188 conservation *via* a systematic kinome-wide structural-based multi-sequence alignment and phylogenetic analysis, and also performed a research survey for reports on the phosphorylation and function of T207/188 equivalents in other EPKs.

**Figure 10:**
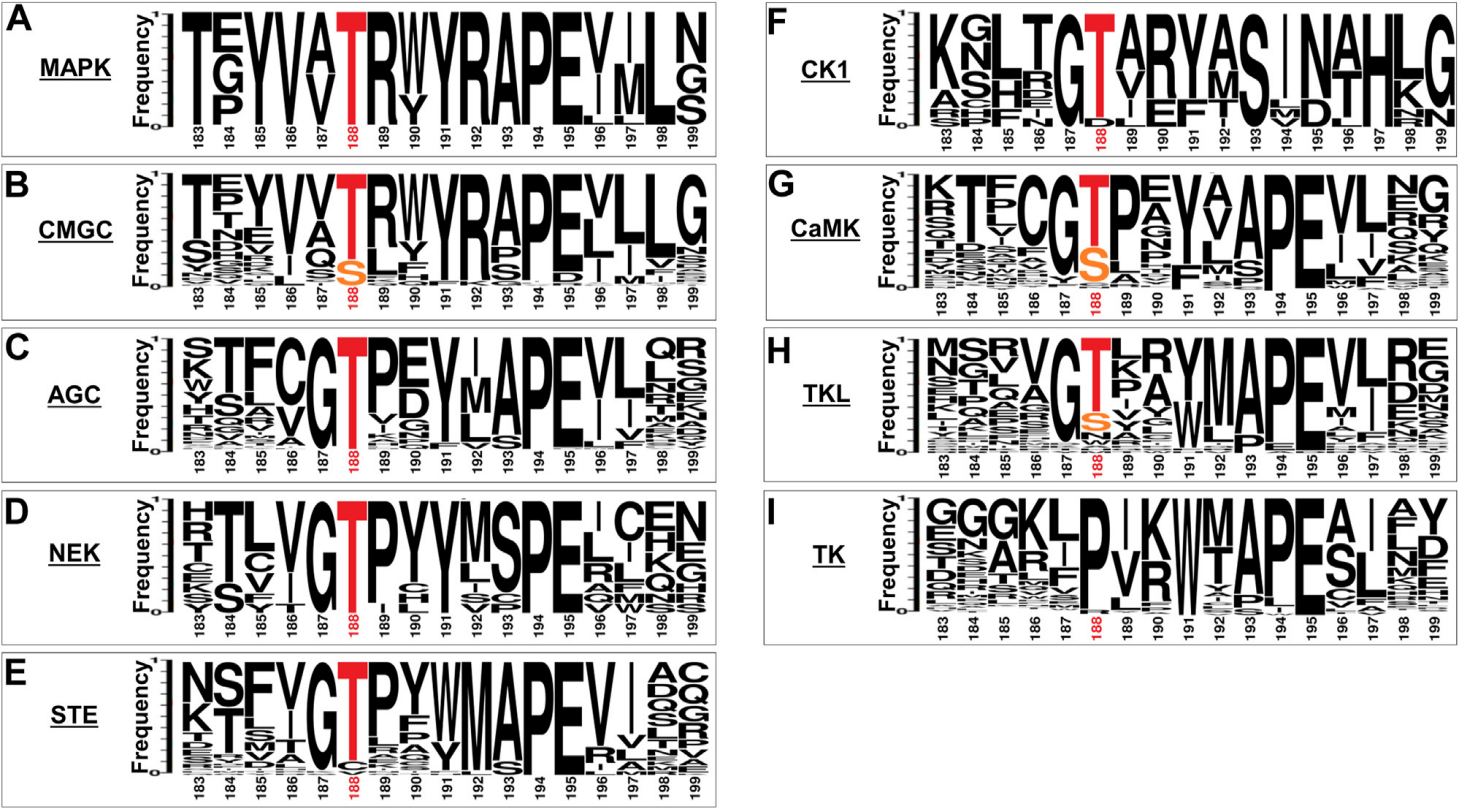
**Erk2 T188-orthologs Ser or Thr are almost invariant in Ser/Thr kinases subfamilies, but totally absent in Tyr kinases subfamily**. Weblogo representation of multiple sequence alignment of the activation loop segment from major kinase groups. The x-axis displays the position of amino acid with numbering of Erk2, and the y-axis represents amino acid frequency.

**Figure 11:**
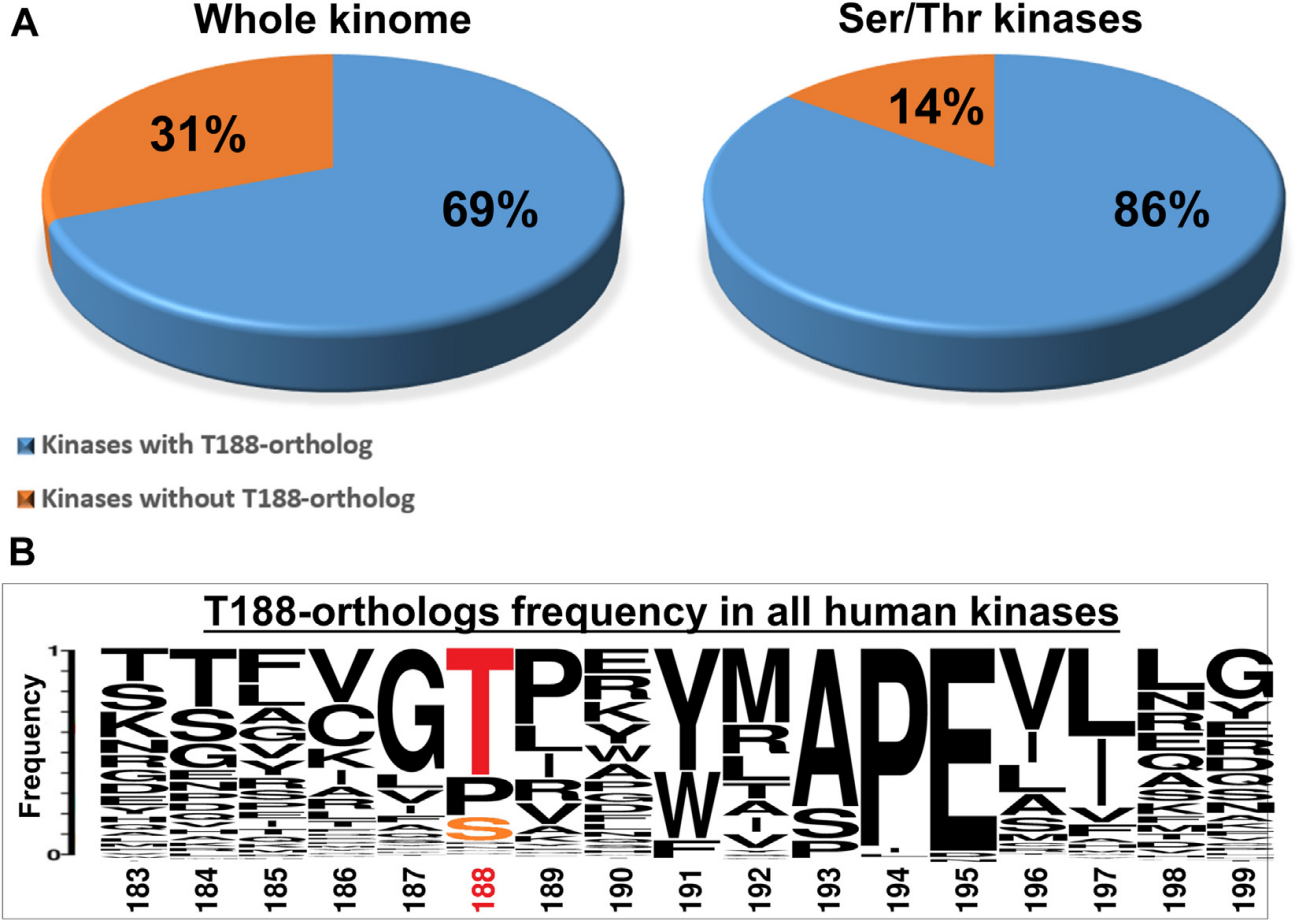
**Erk2 T188-orthologs Ser or Thr are found in 86% of human Ser/Thr kinases.***A,* Graphical representation of Erk2 T188-orthologs frequency in all human kinases (496 sequences), and Ser/Thr kinases (402 sequences). *B*, Weblogo representation of multiple sequence alignment of the activation loop segment of all human kinases. The x-axis displays the position of amino acid with numbering of Erk2, and the y-axis represents amino acid frequency.

As shown in Figure 10*A*, within the family of MAPKs, all kinases with no exception were found to possess a Thr orthologous to Erks' T207/188. Within the CMGC, AGC and NEK families too all kinases, with no exception, were found to possess a T188 ortholog (a Thr or a Ser) (Figs. 10, *B*–*D*; S6 and S7). In the STE subfamily a T188 ortholog is present in 87% of the kinases (Figs. 10*E*, S6 and S7) and in the CK1 subfamily it is present in all kinases except one (Fig. 10*F*; S6 and S7). 92% of CaMK kinases carry a T188 ortholog (Fig. 10*G*, S6 and S7) and 81% of TKL kinases (Fig. 10*H*, S6 and S7). Not a single kinase of the tyrosine kinases group (93 kinases) possesses a T188-ortholog (Fig. 10*I*, S6 and S7). Altogether, 69% of all protein kinases and 86% of Ser/Thr kinases harbor a Thr or a Ser at a position identical to that of Erk's T207/188 (Fig. 11*A*), making it one of the most conserved residues in the activation loop (Fig. 11*B*). Half of the 14% of Ser/Thr kinases that do not possess a T188 ortholog are members of the “Other kinases” group, approximately 50% of which are catalytically inactive.

In summary, a Thr or Ser in the P+1 loop is almost invariant in Ser/Thr kinases. This conservation is particularly significant in light of the fact that residues in its vicinity are less conserved (Fig. 11*B*). An exception is a Gly residue neighboring T207/188, which is highly conserved when analyzing the P+1 sequence of the entire EPK kingdom (Fig. 11), but it is absent from CMGCs, including from MAPKs (Fig. 10) (see Discussion).

### Many EPKs are phosphorylated on the Thr or Ser equivalent to Erks' T207/188

**Figure 12:**
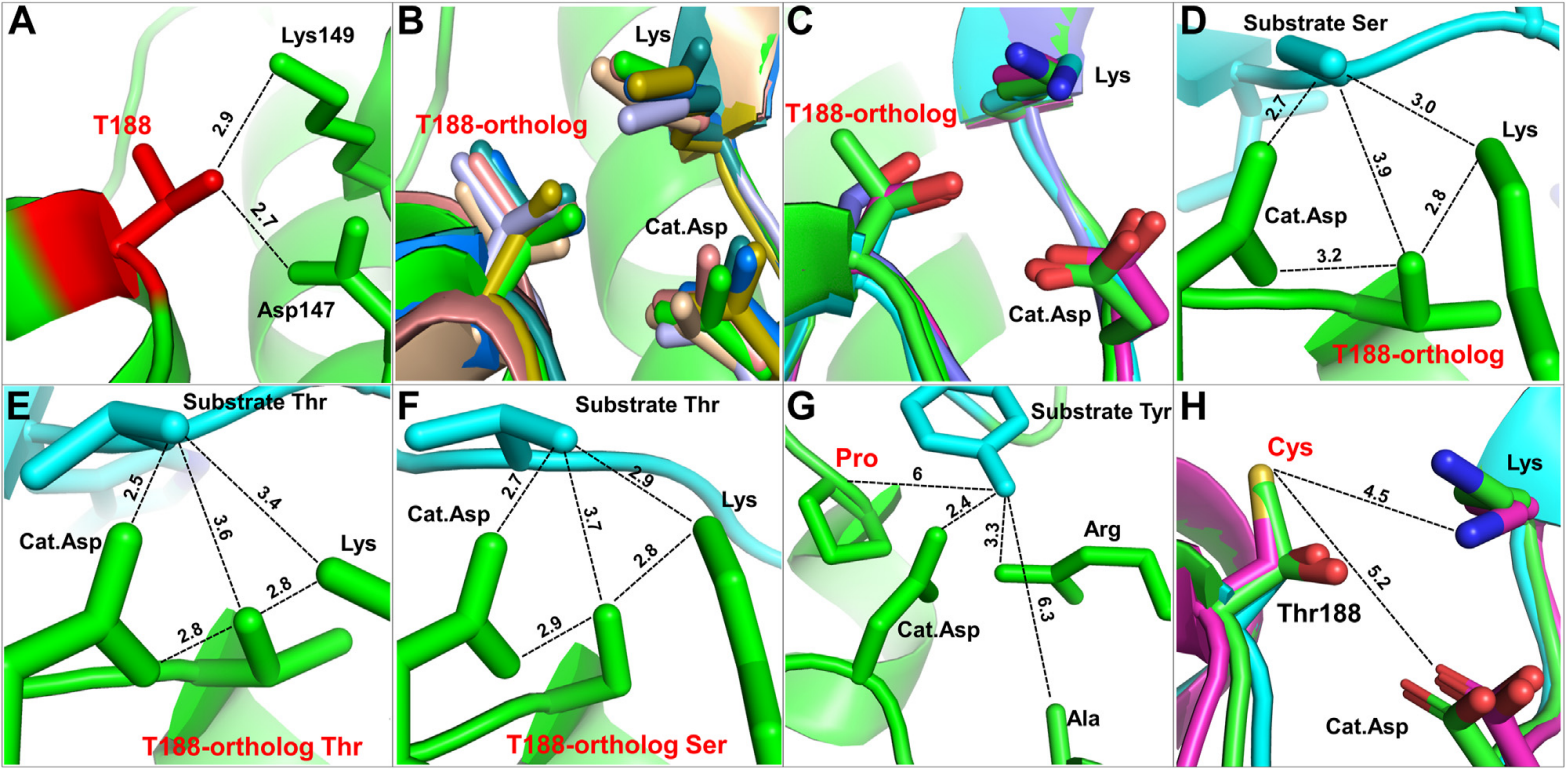
**The spatial arrangement of T188 interactions with the catalytic Asp, Lys, and the substrate's phosphoacceptor sites is conserved in Ser/Thr kinases.** Analysis of crystal structures of representatives from major Ser/Thr kinases subfamilies reveals that in most examined structures T188-orthologs position and association with the catalytic loop and substrates is very similar. *A*, a ribbon representation of inactive human Erk2 (PDB:4S31) demonstrating proximity between T188 and crucial catalytic loop residues Lys149 and Asp147 (catalytic Asp). *B*, superimposition of inactive human Erk2 (PDB:4S31, CMGS, *green*) with catalytic loop and P+1 motif of kinases from other Ser/Thr kinases groups. Structures used were those of PKA (PDB:4WB5, AGC, *marine*), MEKK5 (PDB:2CLQ, STE, *wheat*), TTBK1 (PDB:4NFM, CK1, *olive*), CaMK2B (PDB:3BHH, CaMK, *cyan*), LRRK2 (PDB:7LHW, TKL, *light blue*), STK16 (PDB: 2BUJ, Other kinases, *salmon*). Distances between T188-orthologs, catalytic Asp and Lys in each kinase are provided in Table 2. *C*, examples of analyzed structures possessing T188-orthologs Ser. Superimposition of inactive human Erk2 (PDB:4S31, CMGS, *green*) with catalytic loop and P+1 regions from DYRK1A (PDB:2VX3, CMGS, *cyan*), PRKAA1 (PDB:7JIJ, CaMK, *blue*) and ULK1 (PDB:4WNO, Other kinases, *pink*) all three possessing T188-ortholog Serine. Analysis of kinases structures complexed with substrate revealed that interactions between P+1 loop, catalytic site and substrate are conserved. *D*, Catalytic Subunit of cAMP-dependent Protein kinase complexed with a substrate Peptide (PDB: 1JBP, AGC), T188-ortholog Thr, substrate Ser. *E,* PAK4 in complex with Paktide T peptide substrate (PDB: 4JDH, STE), T188-ortholog Thr, substrate Thr. *F*, DYRK1A in complex with a consensus substrate peptide (PDB: 2WO6, CMGS), T188-ortholog Ser, substrate Thr. *G*, FGFR2 kinase domain in complex with substrate peptide (PDB: 2PVF, TK). Not a single member of Tyr kinases possesses T188-ortholog or adjacent Lys. In all cases there is a Pro at the position equivalent to T188 and an Arg at the position of Lys. *H*, comparison of inactive human Erk2 (PDB:4S31, *green*) with human DYRK1A (PDB:2VX3, *teal*) harboring T188-ortholog Ser and human MAP2K7 (PDB: 5Y90, *pink*) harboring a Cys at the P+1 loop.

The high degree of conservation of the Erks' T207/188 raises the question whether it may also be phosphorylated in other EPKs, as it is in Erk1/2 (Figs. 10 and 12). To address this query, we rigorously searched for papers and phosphoproteomic data that reported phosphorylation of this Thr or Ser, and for studies that mutated it. We found that 160 EPKs were reported to be phosphorylated on T188 orthologs, constituting 41% of Ser/Thr kinases from all major families except RGC (Fig. S7, Table S1). In none of these cases was the effect of phosphorylation on conformation or activity elucidated. Thus, the mode of regulation of Ser/Thr kinases by phosphorylation of the P+1 Thr/Ser seems quite wide, clearly not restricted to Erk1/2. It remains to be revealed whether in other EPKs phosphorylation of the P+1 residue affects phosphorylation of the major activatory phosphoacceptor as is the case for Erk1/2.

**Figure 13:**
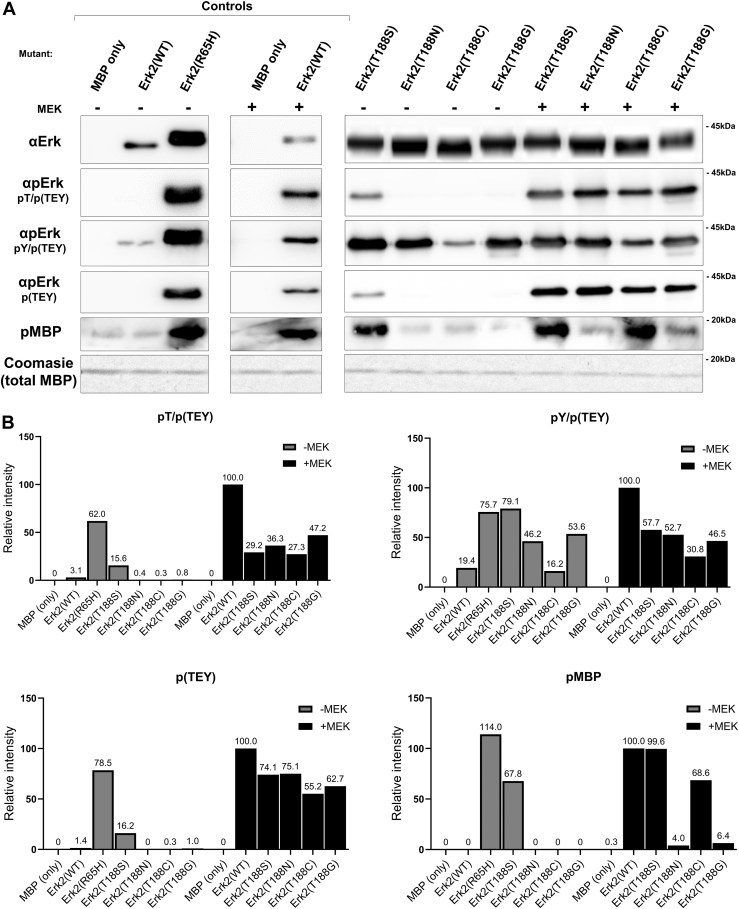
**Erk2^T188S^ and Erk2^T188C^, but not Erk2^T188N^ or Erk2^T188G^, retain catalytic activity *in vitro*.** Catalytic activity toward MBP of the indicated purified proteins was monitored with or without pre-incubation with active MEK1. Substrate's phosphorylation was evaluated using specific anti-phospho-MBP antibodies. *A*, reaction mixtures were subjected to a Western blot analysis with the indicated antibodies. *B*, phosphorylation levels of Erk2 and pMBP were quantified by densitometry (ImageJ) and normalized to total Erk or MBP (-MEK) respectively. Signal of MEK1-activated Erk2^WT^ was defined as 100%.

In addition, we identified reports on 46 kinases in which the residue was mutated and, in all cases, with just one exception, the result was a loss of function (Table 1), as observed for Erk1/2. The single case of T188-ortholog substitution not hampering kinase catalytic activity was mutating T185 of the MAPK p38α to Glycine. Unlike Erks, p38α could be activated not only by MAPK kinases but also by induced autophosphorylation, for example, following binding to TAB1 (35, 36). p38α^T185G^ lost the capability to be induced by TAB1 but maintained the ability to be activated by MAPK kinases (37). In order to evaluate the impact of a similar mutation on Erks, we generated an Erk2^T188G^ molecule and tested the activity of a purified recombinant protein in an *in vitro* kinase assay with MBP as a substrate. This assay demonstrated that in Erk2, substitution of T188 with Gly reduces significantly (to about 6%) MEK-induced catalytic activity *in vitro* (Fig. 13).

### The geometry of T188 interactions with catalytic Asp, neighboring Lys, and substrate's phosphoacceptors is conserved in Ser/Thr kinases

We next asked whether T188 may also be conserved structurally in EPKs and for that looked into its position in currently available crystal structures. As mentioned above (21), T188 of Erk2 is associated with the catalytic/base aspartic acid (D147), with an interaction distance of 2.7 Å (Figs. 1 and 9*A*, Table 2) and with a conserved lysine residue (K149) of a region known as linker 10 (2.9 Å, Figs. 1 and 9*A*, Table 2) (Figs. 12*B*, S8). To assess the conservation of the 3-dimentional structure in the vicinity of T188 we superimposed crystal structures of EPKs representing the seven major families of Ser/Thr kinases (Fig. 12*B*). All of them manifested similar distances and orientations between their orthologs of T188, D147 and K149 (Fig. 12*B*). Superimposition of Erk2 structure with kinases carrying a Ser ortholog of T188 also demonstrated structural conservation of the catalytic loop and the adjacent P+1 loop, containing T188 ortholog (Fig. 12*C*).

**Table 2:** Summary of distances between T188-ortholog and Catalytic Asp and Lys in representatives of major Ser/Thr kinases families

Kinase	Group	PDB	T188-ortholog distance to Lys (Å)	T188-ortholog distance to Cat.Asp (Å)
Erk2	CMGC	4s31	2.9	2.7
PKA	AGC	4wb5	2.8	2.9
MEKK5	STE	2clq	3.3	2.5
TTBK1	CK1	4nfm	2.8	3.5
CAMK2B	CaMK	3bhh	3.2	3.1
LRRK2	TKL	7lhw	2.4	3.4
STK16	Other	2buj	2.8	2.7

Considering the location of T188-orthologs at the P+1 loop, we analyzed crystal structures of substrate-associated kinases (Figs. 12, *D*–*F*). In all cases, orientations, distances, and interactions between four neighboring residues (T188 ortholog, substrate, catalytic Asp and Lys) were very similar. Those results further support the notion of some level of universality regarding the function of T188-ortholog in Ser/Thr kinases. As mentioned, in tyrosine kinases the T188 position is commonly occupied by Pro, and the catalytic loop Lys by Arg (38, 39) (Figs. 12*G*, S8), but the interactions between these residues and the substrate are reminiscent of those observed in Ser/Thr kinases (Fig. 12*G*).

### Erk2^T188S^ and Erk2^T188C^ are catalytically active

Overall, out of 402 Ser/Thr kinases, 290 (72%) possess a Thr at the P+1 loop. 57 Ser/Thr kinases (14%) possess a Ser, 30 of which were found to be phosphorylated. Notably, in nine kinases that contain a Ser equivalent to T188, the Ser was mutated, and in all cases resulted in loss of function (Table 1). Other residues found at this position in some EPKs are Asn (8 kinases) and Cys (7 kinases). Phylogenetic analysis of kinases harboring a Cys at the P+1 site revealed some kinases (STRADA/B of the STE group) that are catalytically inactive (Fig. S6*C*). However, other kinases of the STE group, MAP2Ks consist of kinases harboring a Thr at the P+1 loop (MAP2K1/2/5) and kinases possessing Cys (MAP2K3/4/6/7), and all of them are catalytically active (Fig. S6*C*).

Superimposition of kinase structures possessing Thr (CMGC ERK2), Ser (CMGC DYRK1) or Cys (STE MAP2K7) at P+1 loop (Fig. 12*H*), showed almost identical spatial conformation of all three variants.

As in some catalytically active EPKs the T188 position is occupied by Ser, Asn, or Cys, with structural organization similar to that of Erk2, we asked whether Erk2 may tolerate T188 substitution to Ser, Asn or Cys. Erk2^T188S^, Erk2^T188N^ and Erk2^T188C^ were constructed, purified, and tested in *in vitro* Kinase Assay. Similar to the case of substitution to Ala, Glu, or Asp, substitution of Erk2 T188 to Asn, results in loss of catalytic activity (Fig. 13). However, as proposed by the bioinformatics analysis, substitution of T188 to Ser or Cys did not cause abolishment of catalytic properties. Furthermore, Erk2^T188S^ manifested quite high basal (MEK-independent) activity (Fig. 13). Surprisingly, MEK-induced activity of Erk2^T188C^ mutant is similar to that of Erk2^WT^. It seems that the structural geometry of the region must be very strict and cannot tolerate substitution of T188 to Ala, Glu or Asp, even though this residue is sometimes phosphorylated, probably because Asp and Glu are not at all a perfect mimic of phosphor-Thr. Ser and Cys probably do not affect the delicate structural organization (Fig. 12, *F* and *H*).

## Discussion

Stabilization of EPKs' active conformation through activation loop phosphorylation is common to the vast majority of them. It might be expected, therefore, that the activation loop would be conserved. However, although harboring conserved motifs (*i.e.*, DFG, HRD), the activation segment within the loop is not conserved at the levels of sequence or conformation. It was proposed that this lack of conservation was selected in evolution to render each EPK specific to particular substrate(s) and for acquiring unique properties (40). A few residues stand out from this rule and are more conserved, including two critical threonines. One of those, the “activatory” threonine, has been deeply and widely studied as its phosphorylation leads to stabilizing the active conformation. The possible role of the other threonine, residing 4 to 5 residues C-terminal to the “activatory” one, has not been fully revealed, and its role in Erk1/2 was the subject of this investigation.

As found here, phosphorylation of T207/188 of mammalian Erks occurs in all known variants that acquired auto-activation capability. Also, *in vitro* and *in vivo* T207/188 phosphorylation is not affected by MEKs nor by the main signaling cascade that activates Erks (*i.e.*, not affected by EGF; Figs. S1 and S2, and (8)) (10). It implies that this phosphorylation could be achieved only *via* autophosphorylation, which must be induced, probably allosterically by currently unknown component(s). These allosteric effectors may be more active in skeletal and cardiac muscle than in other tissues and are abnormally overactive in sicknesses of these tissues.

The results obtained here suggest that T207/188 affects the efficiency of the TEY phosphorylation, namely Erk1/2 activation. It could be that by occupying the catalytic Asp, unphosphorylated T207/188 hinders activation of the Thr183 and Tyr185 hydroxyls. Such a latch may be necessary because in the cell, Erks are prone to be phosphorylated as they reside in a complex with MEKs (41). Perhaps in the intrinsically active variants, which all become intrinsically active by self-phosphorylation of the TEY motif, T207/188 phosphorylation causes its repulsion from Asp147, freeing this catalytic Asp to promote activation and consequently phosphorylation of the TEY motif. This notion implies that T207/188 phosphorylation should be independent of TEY phosphorylation and should occur prior to it. Indeed, intrinsically active Erk1/2 molecules that are mutated in the TEY motif (carrying an AEF motif instead) still phosphorylate T207/188 (8, 10). Also, it seems that upon induced expression in *E. coli* T207/188 is phosphorylated first, prior to TEY phosphorylation (Fig. 2, *A* and *B*). This scenario suggests that T207/188 phosphorylation assists TEY autophosphorylation. T207/188 is not phosphorylated when Erks are activated *via* the canonical RTK-Ras-Raf-MEK pathway, suggesting that this phosphorylation is not necessary for activation by MEK1/2. This is because once activated by Raf, MEKs adopt an active conformation, with a strong affinity to the TEY motif. Upon binding to Erk, particularly to the activation segment, probably assisted by scaffold proteins, such as Ksr1 (42) MEKs activate the TEY hydroxyls using their own catalytic Asps. Mutating T207/188 renders TEY more permissive for binding of the MEK1/2/Ksr complex.

Another possible way through which phosphorylation of T207/188 controls TEY phosphorylation is by stabilization of Erks' dimers. Activation of Erks was shown to cause its dimerization (43, 44) and a recent study showed that Erk2^R65S^, which autophosphorylates T188, form a stable dimer spontaneously when expressed in HEK293 cells (45). As Erk dimers can be formed between active and inactive molecules one can suggest a scenario in which a T207/188-phosphorylated monomer functions as an allosteric effector on the other monomer enforces it to autophosphorylate its TEY motif. The two proposed mechanisms are not mutual exclusive as dimerization may assist in shifting the phosho-T207/188 from the catalytic Asp. Maybe in many EPKs phosphorylation of T207/188 equivalent is assisting in autophosphorylation of the activatory threonine by repulsing the threonine residue (or serine in some cases) away from the catalytic Asp. Also, it is known that many kinases form different types of dimers as part of the autophosphorylation reaction (4).

Although the ability to autoactivate exists in Erks, so far it has not been found to occur under physiological conditions. To our knowledge, in all tested systems and experimental models, Erks' activation is dependent on MEKs. This is unambiguously clear in lower eukaryotes, where genetic approaches are easily applied. In yeast (*S. cerevisiae* and *Schizosaccharomyces pombe*), *D. melanogaster* or *Caenorhabditis elegans*, when MEKs are knocked out or suppressed in other ways, Erks are totally inert (22, 33, 46, 47). For other members of the MAPK family, specifically for p38α, p38β and p38γ, physiologically-relevant cases of activation by autophosphorylation were described (35, 36, 48, 49). Perhaps under some physiological conditions, yet unknown, the autophosphorylation activity of Erks is also de-suppressed. It seems that in most mouse tissues de-suppressing mechanisms are constantly active, but at very low levels. Skeletal muscle and cardiac muscle could be the preferred tissues for deciphering the mechanisms that induce phosphorylation of Erks' T207/188. The study of pathological situations that seem to stem from overactivation of Erk *per se, via* the autoactivation mechanisms, including cancers in which the R84H mutations of Erk1 were identified (COSV107307066; cancer.sanger.ac.uk) and cases of cardiac hypertrophy (11) may also assist in resolving the mechanism of Erks' activation *via* T207/188 phosphorylation.

## Experimental procedures

### Plasmids

Plasmids containing cDNAs encoding Erk1^R84S^, Erk1^I103A^, Erk1^S170D^, Erk1^R84S+S170D^, Erk2^Y261A^, Erk2^Y261C^, Erk2^R65S^, Erk2^I84A^, Erk2^S151D^, Erk2^R65S+S151D^, Erk2^Y261A^, Erk2^Y261C^, Erk2^D319N^, Erk2^Y261C^, Mpk1^R68S^, Mpk1^Y268C^, Mpk1^R68S+268C^ were previously described (9, 10, 21).

Previously utilized pET15b and pCEFL vectors, which contain the human 6xHis-tagged ERK1 or HA-tagged rat ERK2 cDNAs (8, 10, 30), served as expression vectors for bacterial and mammalian systems.

For the creation of new mutation pBluescript vectors containing N-terminally 6xHis-tagged human-Erk1^WT^ or HA-tagged rat-Erk2^WT^ or HA-tagged yeast Mpk1^WT^ were used as a template.

### Site-directed mutagenesis

Site-directed mutagenesis was performed according to Agilent QuikChange II Site-Directed Mutagenesis protocol using the PfuUltraII Fusion HS DNA Polymerase (Agilent; catalog no.: 600672). Primers used are listed in Table 3.

**Table 3:** Primers used for site-directed mutagenesis

Description	Primer sequence
Erk1-P75G-F	5′- CCATCAAGAAGATCAGCGGCTTCGAACATCAGACCTA -3′
Erk1-P75G-R	5′- TAGGTCTGATGTTCGAAGCCGCTGATCTTCTTGATGG -3′
Erk1-P75L-F	5′- CCATCAAGAAGATCAGCCTCTTCGAACATCAGACCTA -3′
Erk1-P75L-R	5′- TAGGTCTGATGTTCGAAGAGGCTGATCTTCTTGATGG -3′
Erk1-E98K-F	5′- TGCTGCGCTTCCGCCATAAGAATGTCATCGGCATCCG -3′
Erk1-E98K-R	5′- CGGATGCCGATGACATTCTTATGGCGGAAGCGCAGCA -3′
Erk1-S159L-F	5′- CCTCAAGTACATCCACTTGGCCAACGTGCTCCACC -3′
Erk1-S159L-R	5′- GGTGGAGCACGTTGGCCAAGTGGATGTACTTGAGG -3′
Erk1-T207A-F	5′- GAGTATGTGGCTGCGCGCTGGTACCGGG-3′
Erk1-T207A-R	5′- CCCGGTACCAGCGCGCAGCCACATACTCC -3′
Erk1-T207E-F	5′- GGAGTATGTGGCTGAGCGCTGGTACCGGG -3′
Erk1-T207E-R	5′- CCCGGTACCAGCGCTCAGCCACATACTCC -3′
Erk1-E207D-F	5′- GAGTATGTGGCTGACCGCTGGTACCGGG -3′
Erk1-E207D-R	5′- CCCGGTACCAGCGGTCAGCCACATACTCC -3′
Erk1-R84H-F	5′- CAGACCTACTGCCAGCACACGCTCCGGGAGATC -3′
Erk1-R84H-R	5′- GATCTCCCGGAGCGTGTGCTGGCAGTAGGTCTG -3′
Erk1-G184D-F	5′- TAAGATTTGTGATTTCGACCTGGCCCGGATTGCC -3′
Erk1-G184D-R	5′- GGCAATCCGGGCCAGGTCGAAATCACAAATCTTA -3′
Erk1-P75G-F	5′- CCATCAAGAAGATCAGCGGCTTCGAACATCAGACCTA-3′
Erk1-P75G-R	5′- TAGGTCTGATGTTCGAAGCCGCTGATCTTCTTGATGG-3′
Erk1-P75L-F	5′- CCATCAAGAAGATCAGCCTCTTCGAACATCAGACCTA-3′
Erk1-P75L-R	5′- TAGGTCTGATGTTCGAAGAGGCTGATCTTCTTGATGG-3′
Erk1-E98K-F	5′- TGCTGCGCTTCCGCCATAAGAATGTCATCGGCATCCG-3′
Erk1- E98 K -R	5′- CGGATGCCGATGACATTCTTATGGCGGAAGCGCAGCA-3′
Erk1-S159L-F	5′- CCTCAAGTACATCCACTTGGCCAACGTGCTCCACC-3′
Erk1-S159L-R	5′- GGTGGAGCACGTTGGCCAAGTGGATGTACTTGAGG-3′
Erk2-P56G-F	5′- GCTATCAAGAAAATCAGTGGTTTTGAGCACCAGACCTAC -3′
Erk2-P56G-R	5′- GTAGGTCTGGTGCTCAAAACCACTGATTTTCTTGATAGC -3′
Erk2-P56L-F	5′- GCTATCAAGAAAATCAGTCTTTTTGAGCACCAGACCTAC -3′
Erk2-P56L-R	5′- GTAGGTCTGGTGCTCAAAAAGACTGATTTTCTTGATAGC -3′
Erk2-E79K-F	5′- CTGCGCTTCAGACATAAGAACATCATCGGCATC -3′
Erk2-E78K-R	5′- GATGCCGATGATGTTCTTATGTCTGAAGCGCAG -3′
Erk2-S139L-F	5′- GAGGATTAAAGTATATACATTTAGCTAATGTTCTGCACCGTG -3′
Erk2-S139L-R	5′- CACGGTGCAGAACATTAGCTAAATGTATATACTTTAATCCTC -3′
Erk2-T188A-F	5′- CAGAGTATGTAGCCGCGCGTTGGTACAGAGC -3′
Erk2-T188A-R	5′- GCTCTGTACCAACGCGCGGCTACATACTCTG -3′
Erk2-T188E-F	5′- GACAGAGTATGTAGCCGAGCGTTGGTACAGAGCTC -3′
Erk2-T188E-R	5′- GAGCTCTGTACCAACGCTCGGCTACATACTCTGTC -3′
Erk2-E188D-F	5′- CAGAGTATGTAGCCGACCGTTGGTACAGAGC -3′
Erk2-E188D-R	5′- GCTCTGTACCAACGGTCGGCTACATACTCTG -3′
Erk2-K65Y-F	5′- GCACCAGACCTACTGTCAGTATACCCTGAGAGAGATAAAAA -3′
Erk2-K65Y-R	5′- TTTTTATCTCTCTCAGGGTATACTGACAGTAGGTCTGGTGC -3′
Erk2-Y65H-F	5′- GCACCAGACCTACTGTCAGCATACCCTGAGAGAGATAAAAA -3′
Erk2-Y65H-R	5′- TTTTTATCTCTCTCAGGGTATGCTGACAGTAGGTCTGGTGC -3′
Erk2-G167D-F	5′- CAAGATCTGTGACTTTGACCTTGCCCGTGTTGCAG -3′
Erk2-G167D-R	5′- CTGCAACACGGGCAAGGTCAAAGTCACAGATCTTG -3′
Erk2-P56G-F	5′- GCTATCAAGAAAATCAGTGGTTTTGAGCACCAGACCTAC-3′
Erk2-P56G-R	5′- GTAGGTCTGGTGCTCAAAACCACTGATTTTCTTGATAGC-3′
Erk2-P56L-F	5′- GCTATCAAGAAAATCAGTCTTTTTGAGCACCAGACCTAC-3′
Erk2-P56L-R	5′- GTAGGTCTGGTGCTCAAAAAGACTGATTTTCTTGATAGC-3′
Erk2-E79K-F	5′- CTGCGCTTCAGACATAAGAACATCATCGGCATC-3′
Erk2-E79K-R	5′- GATGCCGATGATGTTCTTATGTCTGAAGCGCAG-3′
Erk1-S140L-F	5′-GAGGATTAAAGTATATACATTTAGCTAATGTTCTGCACCGTG-3′
Erk1-S140L-R	5′-CACGGTGCAGAACATTAGCTAAATGTATATACTTTAATCCTC-3′
Erk2-T188S-F	5′- CAGAGTATGTAGCCTCGCGTTGGTACAGAGCTCC-3′
Erk2-T188S-R	5′- GGAGCTCTGTACCAACGCGAGGCTACATACTCTG -3′
Erk2-T188N-F	5′- CAGAGTATGTAGCCAACCGTTGGTACAGAGCTCC -3′
Erk2-T188N-R	5′- GGAGCTCTGTACCAACGGTTGGCTACATACTCTG -3′
Erk2-T188C-F	5′- CAGAGTATGTAGCCTGCCGTTGGTACAGAGCTCC -3′
Erk2-T188C-R	5′- GGAGCTCTGTACCAACGGCAGGCTACATACTCTG -3′
Erk2-T188G-F	5′- CTTGACAGAGTATGTAGCCGGGCGTTGGTACAGAGCTCC -3′
Erk2-T188G-R	5′- GGAGCTCTGTACCAACGCCCGGCTACATACTCTGTCAAG -3′

### Protein purification

Proteins were expressed in Rosetta *E. coli* cells in Lysogeny broth (LB) liquid media with 100 μg/ml ampicillin and 50 μg/ml chloramphenicol. Cultures were incubated overnight at 37 °C in 6 ml media. The following day each starter was added to 250 ml of LB media with the antibiotics and incubated for 3 to 4 h at 37 °C. After OD_600_ reached 0.3 to 0.4 protein expression was induced by adding 0.3 mM IPTG and incubating another 5 h at 30 °C. Cells were pelleted by centrifugation at 3200 rcf for 10 min, sonicated in buffer containing 0.3 M NaCl, 50 mM Tris-HCl pH = 8, 250 mM Imidazole, pelleted again at 3200 rcf for 10 min and stored in −70 °C. Frozen cells were thawed on ice and washed in cold buffer containing 0.3 M NaCl, 50 mM Tris-HCl pH = 8, 250 mM Imidazole and the protein inhibitors: 2.3 μM Leupeptine, 1.45 μM Pepstatin, 0.15 μM Aprotinin, 520 μM Benzamidine and 100 μM PMSF. Cells were disrupted by sonicator at 30% amplitude, three sets of 10 s pulse following 10 s rest, twice with 1-min rest on ice in between. Cell lysates were pelleted by centrifugation at 20,000 rcf for 40 min at 4 °C. As all recombinant proteins contained His-tag purification was performed on Nickel-nitrilotriacetic acid agarose column. First, lysates were loaded into the column and flow through it. After column was washed using buffer containing 0.3 M NaCl, 50 mM Tris-HCl pH = 8, 250 mM Imidazole, proteins were eluted with a mixture of 0.3 M NaCl, 50 mM Tris-HCl pH = 8, and 250 mM Imidasole. Eluted proteins were collected in fractions of 1 ml. Overnight dialysis was done in buffer containing 12.5 mM HEPES pH = 7.5, 14.9 mg/ml KCl, 0.5 mM DTT and 6.25% Glycerol. Fractions were divided to smaller aliquots, frozen in liquid nitrogen and stored at −70 °C.

Protein purification level was determined by gel electrophoresis on 12% polyacrylamide SDS-PAGE.

### Protein quantification

Protein quantification was done using Machery-Nagel Protein Quantification Assay Kit, Microplate assay procedure protocol and confirmed with densitometry in ImageJ.

### Kinase assay

*In vitro* kinase assay was performed with MBP as a substrate. In some cases [γ-^32^P]ATP was included and phosphorylation of substrate was measured by incorporation of 32p. In other cases, MBP phosphorylation was monitored by specific anti-phosMBP antibodies. In radioactive kinase assay, when Mek-activated Erks were tested 1 μg of purified recombinant protein was activated by 12 ng of activated Mek1 for 30 min at 30 °C. Reaction conditions were as follows: 150 mM NaCl, 50 mM Tris-Cl (pH = 8), 75 mM MgCl_2_, 25 mM β-glycerol phosphate, 0.1 mM dithiothreitol (DDT), 1 mM Na_3_VO_4_, 5 mM ethylene glycol tetra acetic acid (EGTA) and 0.5 mM ATP. Triplicates of 5 μl from initiated reaction (on ice) were incubated with 45 μl of kinase reaction buffer (KRB) for 15 min at 30 °C. Reaction was terminated by addition of 50 μl EGTA and placing on ice. Final kinase reaction buffer concentration were: 20 mM HEPES pH = 8.0, 0.1 mM Benzamidine, 0.1 mM dithiothreitol (DTT), 10 mM MgCl_2_, 0.1 mM ATP, 0.5 μg/μl MBP, 25 mM β-glycerol phosphate, 1 mM Na_3_V0_4_ and 0.1 μCi of [γ-^32^P]ATP. At the end of the reaction 85 μl were spotted on 3 × 3 cm Whatman paper squares and air-dried for 1h. Each Whatman square was then washed 4 times in 10% TCA and 3% sodium pyrophosphate, 3 times for 1.5 h and one overnight. Next day paper squares were rinsed twice in 100% ethanol for 20 min each time, and air-dried. Radioactivity of each paper square was measured using scintillation counter running a ^32^P Cherenkov program. In addition, 15 μl of each reaction was boiled at 100 °C for 5 min with 5 μl Laemmli sample buffer (x4) and separated on 12% SDS-PAGE. Gel was stained with Coomassie for 20 min, and de-stained overnight using solution of 20% methanol with 10% Acetic acid. After evaluation of the substrate, gel was dried and exposed to X-Ray film and phosphor screens for Amersham Typhoon Biomolecular Imager. In non-radioactive kinase assay, when Mek-activated molecules were tested 3.5 μg of purified recombinant protein were activated by 30 ng activated Mek1 for 30 min at 30 °C. Reaction conditions were as follows: 150 mM NaCl, 50 mM Tris-Cl (pH = 8), 75 mM MgCl_2_, 25 mM β-glycerol phosphate, 0.1 mM dithiothreitol (DDT), 1 mM Na_3_VO_4_, 5 mM ethylene glycol tetra acetic acid (EGTA) and 0.5 mM ATP. 20 μl from initiated reaction were incubated with 180 μl of kinase reaction buffer (KRB) for 30 min at 30 °C. Final kinase reaction buffer concentration was: 20 mM HEPES pH = 8.0, 0.1 mM Benzamidine, 0.1 mM dithiothreitol (DTT), 10 mM MgCl_2_, 0.5 mM ATP, 25 mM β-glycerol phosphate, 1 mM Na_3_V0_4_ and 25 ng/μl MBP. Reaction was terminated by addition of 50 μl Laemmli sample buffer (x4) and boiled at 100 °C for 10 min 15 μl from each sample was separated on 12% SDS-PAGE and analyzed by Western blot with the indicated antibodies. Gels were stained with Coomassie for 10 min, and de-stained overnight in solution of 20% methanol with 10% Acetic acid was used to evaluate total amount of MBP substrate.

### Western blotting

Western blotting was performed as previously described (10). Primary antibodies used are specified in Table 4.

**Table 4:** Antibodies used in Western blot analysis

Antibody	Manufacturer	Catalog number
Erk	Cell Signaling	4695
pErk pT/p(TEY)	Cell Signaling	4370
pErk pY/p(TEY)	Cell Signaling	4377
pErk p(TEY)	Sigma-Aldrich	M8159
GAPDH	ThermoFisher	MA515738
pT207/188	Kinexus Bioinformatics	PK865
HA	Roche	11867423001
His	Sigma-Aldrich	H1029
CDC2	Santa Cruz Biotechnology	SC-53
pMBP	Sigma-Aldrich	05-429

### Mass spectrometry

#### Sample preparation for MS analysis

Proteins were denatured in 100 μl 8M urea, 10 mM DTT, 25 mM Tris-HCl pH 8.0, for 30 min. Iodoacetamide (50 mM final concentration) was added and proteins were further incubated for 30 min in the dark. The Urea was diluted to 1 M using 25 mM Tris-HCl pH 8.0. Trypsin was added (0.4 μg/sample) and incubated overnight at 37 °C with gentle agitation. Next day the peptides were acidified using formic acid to final concentration of 0.38% and desalted on C18 home-made Stage tips.

#### Nano LC-MS/MS analysis

MS analysis was performed using a Q Exactive HF mass spectrometer (Thermo Fisher Scientific) coupled on-line to a nanoflow UHPLC instrument, Ultimate 3000 Dionex (Thermo Fisher Scientific). Peptides (0.1 μg, as estimated by O.D._280_ nm) were separated over a 62 min gradient (3–50% acetonitrile) run at a flow rate of 0.3 μl/min on a reverse phase 25-cm-long C18 column (75 μm ID, 2 μm, 100 Å, Thermo PepMapRSLC) for 120 min. The survey scans (350–1500 m/z, target value 3E6 charges, maximum ion injection times 100 ms) were acquired and followed by higher energy collisional dissociation (HCD) based fragmentation (normalized collision energy 30). A resolution of 120,000 was used for survey scans and up to 15 dynamically chosen most abundant precursor ions from an inclusion list were chosen for fragmentation (isolation window 4.0 m/z). The MS/MS scans were acquired at a resolution of 45,000 (target value 5E5 charges, maximum ion injection times 50 ms). Dynamic exclusion was 10 s. Data were acquired using Xcalibur software (Thermo Scientific). To avoid a carryover, the column was washed with 80% acetonitrile, 0.1% formic acid for 25 min between samples.

#### MS data analysis

The data analysis was performed using MetaMorpheus version 1.0.5. Raw data was searched against the sequences of MEK1 (uniport ID), ERK2 (uniport ID), BSA (uniport ID) and all modified proteins that were used, in addition to a list of common contaminants. Calibration and search tasks were run using all default settings, GPTMD task was used for phosphorylation (STY) detection.

### Yeast cell plasmid transformation

Yeast transformations were performed by lithium acetate method as previously described (50).

### Growth assay of yeast cells (Drop assay)

Yeast cultures of the *mpk1Δ* and *mkk1Δmkk2Δ* strains (10), harboring plasmids, were grown in YNB(–URA) liquid medium (0.17% yeast nitrogen base without amino acid and ammonium sulfate, 0.5% NH_4_(SO_4_)_2_, 2% glucose and the required amino acids and nitrogen bases, but with no uracil) until they reached mid-logarithmic phase (OD_600_ = 0.5). Five serial 10-fold dilutions were then prepared, starting from an OD_600_ of 0.4 (with approximate cell concentrations of 10^7^, 10^6^, 10^5^, 10^4^, and 10^3^ cells/ml). 5 μl of each dilution were spotted onto plates containing YPD (2% glucose, 1% yeast extract and 2% bacto peptone) supplemented with 15 mM caffeine or onto plates containing YNB(–URA) medium. The plates were incubated at 30 °C for 6 days.

### Mammalian cell culture transfection

NIH3T3 cells were grown in DMEM supplemented with 10% fetal bovine serum (FBS), 0.044 M sodium bicarbonate, and streptomycin and penicillin (Beit-Haemek). HEK293T cells were cultured in the same medium but with 10% FBS, 0.017 M sodium bicarbonate, 1 mM sodium pyruvate, and streptomycin and penicillin. All cells were incubated at 37 °C with 5% CO_2_. HEK293T cells were plated 24 h before transfection at densities of 1.5 × 10^5^ cells per 3-cm plate or 8 × 10^5^ cells per 10-cm plate. The medium was changed to half the volume approximately 2 h before transfection. For transfection, 10 μg of cDNA (pCEFL plasmids) was used for 24 h *via* the calcium phosphate method (51). After transfection, cells were washed with phosphate-buffered saline (PBS) and supplied with fresh medium. At 24 h post-transfection, cells were starved for 16 h by incubation in serum-free medium and exposed or not exposed to 50 ng/ml EGF for 10 min. Cells were then lysed with 80 μl of sample buffer containing 10% glycerol, 3% SDS, 0.2 M Tris (pH 6.8), 5% β-mercaptoethanol, and phenol blue dye, and boiled at 100 °C for 10 min. NIH3T3 cells were transfected using TurboFect Transfection Reagent (Thermo Scientific).

### Foci formation assay

NIH3T3 cells were transfected using TurboFect transfection reagent (Thermo Scientific; catalog no.: R0533) as per the manufacturer's instructions. 48 h after transfection, cells were selected for plasmid presence by treatment with 400 μg/ml G-418 (Sigma-Aldrich; catalog no.: A1720). Four weeks following transfection, the cells were washed with PBS, fixed in 100% methanol for 20 min, and stained with 4% crystal violet dye for 5 min.

### Mouse tissues extraction and preparation

#### Mice

The protocols for mouse breeding and research, NS-19 to 15,807-4, were approved by the IACUC of the Hebrew University of Jerusalem.

#### Anesthesia

Mice were administered an overdose of Ketamine + Medetomidine/Saline cocktail (0.75 mg/ml Ketamine + 0.1 mg/ml Medetomidine) *via* intraperitoneal injection, using 0.015 ml per gram of body weight. Following the injection, cardiac puncture was performed to collect tissues. The collected tissues were then processed and/or stored for analysis as described below.

#### Protein lysate preparations

Protein extracts from the organs of mice were prepared using liquid nitrogen-frozen tissues. The tissues were lysed in 2 × tissue volume lysis buffer (50 mM HEPES, pH 7.5, 150 mM NaCl, 1 mM EDTA, 1% Triton X-100, 0.1% sodium deoxycholate, 0.1% SDS), supplemented with HALTTM Protease and Phosphatase Inhibitor Cocktail (#78440; Thermo Fisher Scientific). Tissues were homogenized with a Bullet Blender Tissue Homogenizer and the appropriate beads (1 × tissue volume, Next Advance) following the manufacturer's protocol. The homogenized supernatant was collected, mixed with 2 × SDS sample buffer, and boiled for 10 min. Protein quantification was carried out using the MN Protein Quantification Assay Reagents Kit (740,967.250, MACHEREY-NAGEL).

### Human tissues preparation

Human biopsies were used under permission of the Helsinki committee of the Hadassah-Hebrew University medical center, protocol #0555-21-HMO. Fresh frozen skeletal muscle samples were obtained by standard open biopsy from quadriceps, biceps or deltoid of patients suffering of myopathies/myosotis. Samples were washed with PBS(X1) to remove OCT (optimal cutting temperature embedding medium) and protein lysates for Western Blots were prepared as previously described (see: Mouse tissues extraction and preparation**).**

### AlphaFold

The model of Erk2 protein phosphorylated on T188 was computationally predicted based on their amino acid sequences using the AlphaFold 3 Server (accessed on 25/05/2024) (34).

## Data availability

Data are be shared upon request.

Contact information: Alexey Baskin (alexey.baskin@mail.huji.ac.il)

## Supporting information

This article contains supporting information.

## Conflict of interest

The authors declare that they have no conflicts of interest with the contents of this article.
