# Supplementary material for: All intrinsically active Erk1/2 mutants autophosphorylate threonine207/188, a plausible regulator of the TEY motif phosphorylation
Source: J Biol Chem. 2025 Apr 11;301(6):108509. doi: 10.1016/j.jbc.2025.108509 (PMC12141571; doi:10.1016/j.jbc.2025.108509)
Supplement: Supporting information [file mmc1.pdf]

Supporting Information

All intrinsically active Erk1/2 mutants autophosphorylate Threonine207/188, a plausible regulator of the TEY motif phosphorylation

Baskin et.al

This PDF file include:

1. Supplementary results
2. Supplementary figures 1-8
3. Supplementary Table

Supplementary results:

Erk1/2 variants, reported as gain-of-function mutants, are not intrinsically active catalytically and are not phosphorylated on T207/188

As all currently known Erk1/2 mutants that were proven to be *bona fide* intrinsically catalytically active do autophosphorylate T207/188, we checked whether other variants of Erks, reported to be active, may also autophosphorylate this residue.

An array of Erk2 mutants considered to be overactive has been reported by Brenan et al. (108). These authors introduced a library of randomly mutated Erk2 molecules to A375 cells and selected for mutants that affected cell proliferation. Mutants that enhanced proliferation were considered loss-of-function mutants, while mutants that imposed slower growth were considered GOF mutants (108). Prominent GOF mutants (on the basis of this criterion) were E78K, P55G, P55L, S140L, D319V, D319N, E320K and E320V. In another screen Brenan et al. found that any of 11 different substitutions at Pro55, or the mutation G167D, makes Erk2 resistant to the pharmacological inhibitors VRT-11E and SCH772984 (108). An equivalent mutation in Erk1, G184D, was also identified in another study to render the kinase resistant to inhibitors (109). The G184D/G165D mutation is intriguing as G184/165 is part of the DFG motif, critical for binding Mg^{2+}/ATP.

Mutations in DFG are known to reduce catalytic activity, rather than to improve it or to render it resistant to inhibitors (110, 111).

Of the Erk2 GOF mutations reported by Brenan et al., those occurring in D319 and E320 are equivalent to a previously identified GOF mutation, known as *sevenmaker*, in the *Drosophila's* Erk. Their underlying mechanism is a reduction in affinity to phosphatases rather than elevation of intrinsic activity (112, 113); reviewed in (114, 115)), and are therefore not in the interest of this study. For the other proposed GOF mutants it is not known whether they are intrinsically active catalytically, phosphorylated on T207/188 and/or acquired oncogenic capabilities. To test these queries we mutated ERK1/2 in P75/56, E98/79, S159/140 and G184/165, and assessed the resulting mutants in four assays: 1) Measuring catalytic activity in an *in vitro* kinase assay of purified recombinant proteins. 2) Monitoring TEY and T207/188 phosphorylation levels. 3) Monitoring spontaneous activity following transient expression in cells in culture. 4) Testing oncogenic activity by monitoring appearance of transformed foci following expression in NIH3T3 cells.

In vitro kinase assays revealed that none of the putative GOF mutants tested manifested any intrinsic (MEK-independent) activity (Fig. S1). They behaved similar to Erk1/2^{WT}. In fact, when assayed for MEK-induced catalysis some the mutants showed rather lower activity than Erk1/2^{WT}. Erk1^{P75L} manifested only 40% of the activity shown by Erk1^{WT}, and Erk1^{S159L} 62% (Fig. S1A). The activity of MEK-phosphorylated Erk1^{G184D} was only 29% of the activity of MEK- phosphorylated Erk1^{WT} (Fig. S1B) and that of Erk2^{G165D} 36% (Fig. S2D). The reduced activity of these latter mutants is expected as they are mutated in the DFG motif, but is intriguing, as the G184D/G165D mutations are considered important because they render Erks resistant to inhibitors that are in clinical trials (109, 116). We thus further tested this mutation on the background of intrinsically active variants that may occur in cancer, Erk1^{R84S} and Erk2^{R65S} (114). Kinase activity of recombinant Erk1 $^{R84S+G184D}$ and Erk2$^{R65S+G165D}$ was extremely low, ~3% of the activity of Erk1/2^{WT} (Figs. S1B and S1D). These observations bring into question the significance of the drug-resistance caused by the G184D/G165D mutation as the mutation anyway causes a significant reduction in activity.

In accordance with the lack of intrinsic activity, none of the mutants was spontaneously phosphorylated on the TEY motif, showing that they did not acquire an autophosphorylation capability (Fig. S1). They were similarly not phosphorylated on T207/188 (Figs. S1 and S2).

In line with the observations *in vitro*, when expressed in HEK293 cells all mutants were not spontaneously active, as reflected by the lack of phosphorylation of the TEY motif. They were

phosphorylated, just as Erk1/2^{WT}, in response to EGF (Fig. S2). Finally, none of the mutants showed oncogenic capability as not a single focus appeared following their expression in NIH3T3 cells (Fig. S3).

Thus, T207/188 phosphorylation, which is associated with intrinsic autophosphorylation capability, does not occur in the proposed GOF mutants (108) tested here. It seems restricted to Erk1/2 mutants that gained a *bona fide* intrinsic catalytic capability.

Supplementary figures:

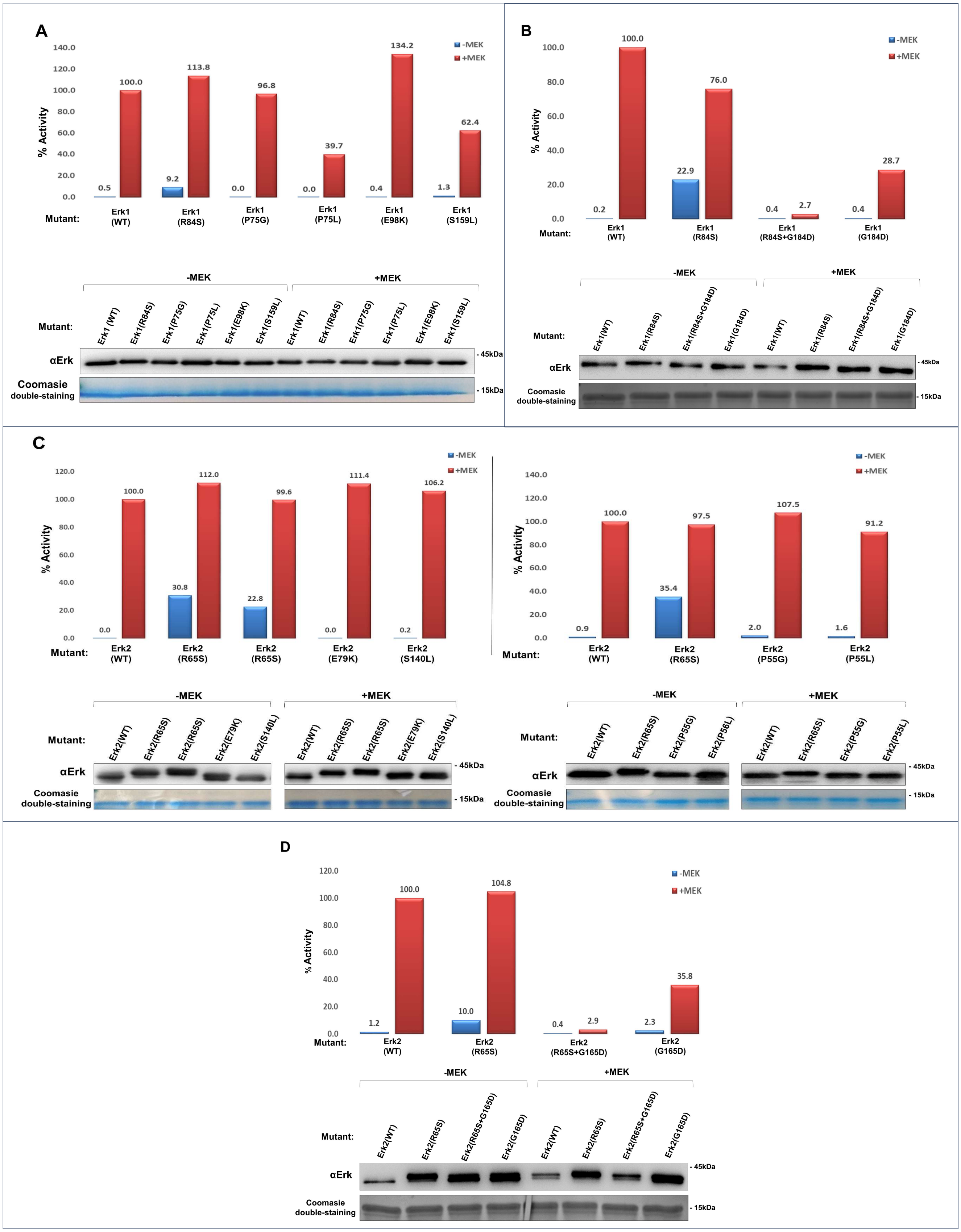

Figure S1. Reported gain-of-function mutants of Erk1/2 are not inherently catalytically active. Effect of GOF mutations reported by Brenan et al., (1) and Jha et al., (2) on intrinsic and MEK-induced catalytic activity on Erk1 (A, B) and Erk2 (C, D) was analyzed. Activity of the indicated purified proteins was monitored with or without pre-incubation with active MEK1, using [γ-^{32}p]ATP and MBP as substrates. Reaction mixtures were spotted on filter papers and quantified. Activity of MEK1-activated Erk1/2^{WT} was defined as 100%. In parallel, a sample from each reaction was subjected to a Western blot analysis with the antibodies against total Erk1/2 and to SDS-PAGE following by Coomassie blue staining to visualize amounts of the MBP substrate. Reactions were performed in triplicates.

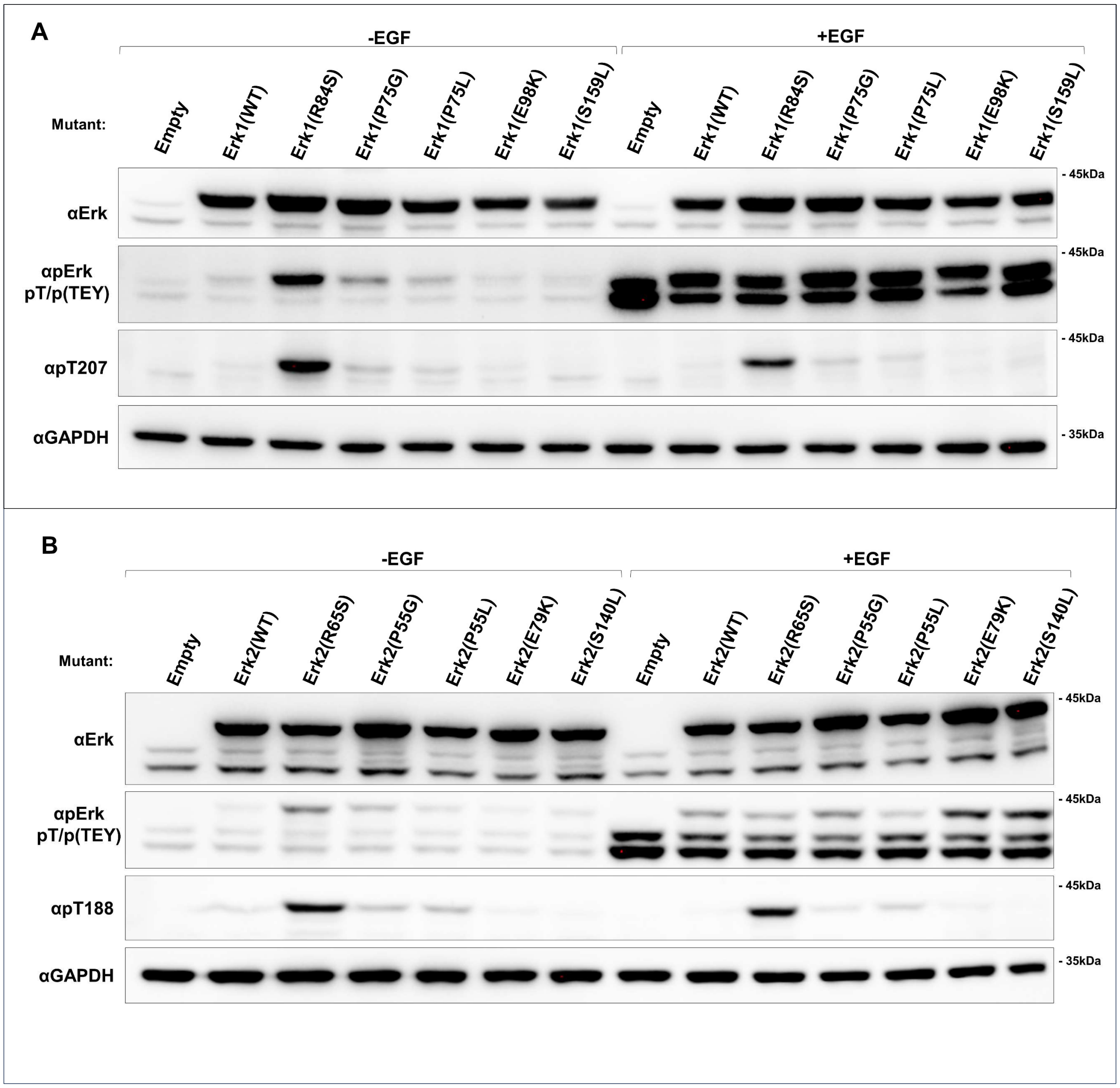

Figure S2. Reported gain-of-function mutants of Erk1/2 are not phosphorylated at T207/188. HEK293T cells were transfected with pCEFL vectors containing the specified Erk1 (A) or Erk2 (B) mutants. After 48 hours the cells were serum-starved for 16 hours and subsequently treated (+) or left untreated (-) with EGF (50 ng/ml for 10 minutes). Cell lysates were then prepared and analyzed by Western blot using the indicated antibodies.

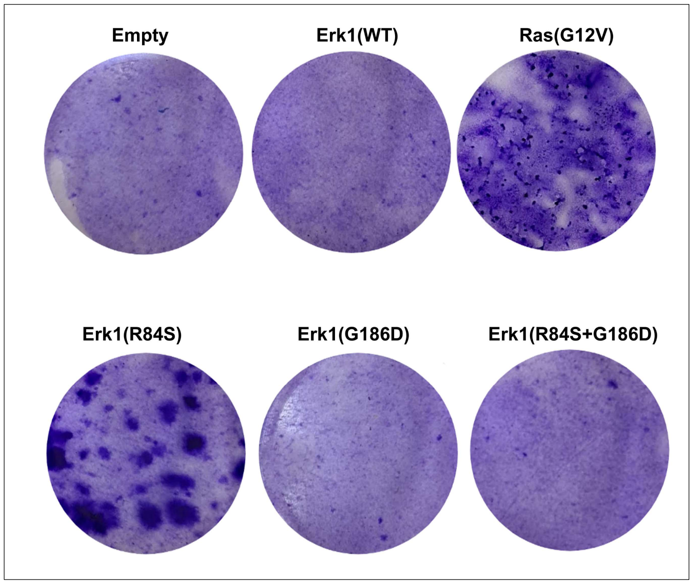

Figure S3. Mutating G184 of Erk1 to D (a mutation reported to confer resistant to Erk's pharmacological inhibitors) abolished the oncogenic capability of Erk1^{R84S}. NIH3T3 cells were transfected with expression vectors carrying the cDNAs encoding Ha-Ras^{Val12} or the indicated Erk1 molecules. Selection of cells harboring the vector was achieved by addition of G418. Cells were fixed and stained with crystal violet 4 weeks after transfection.

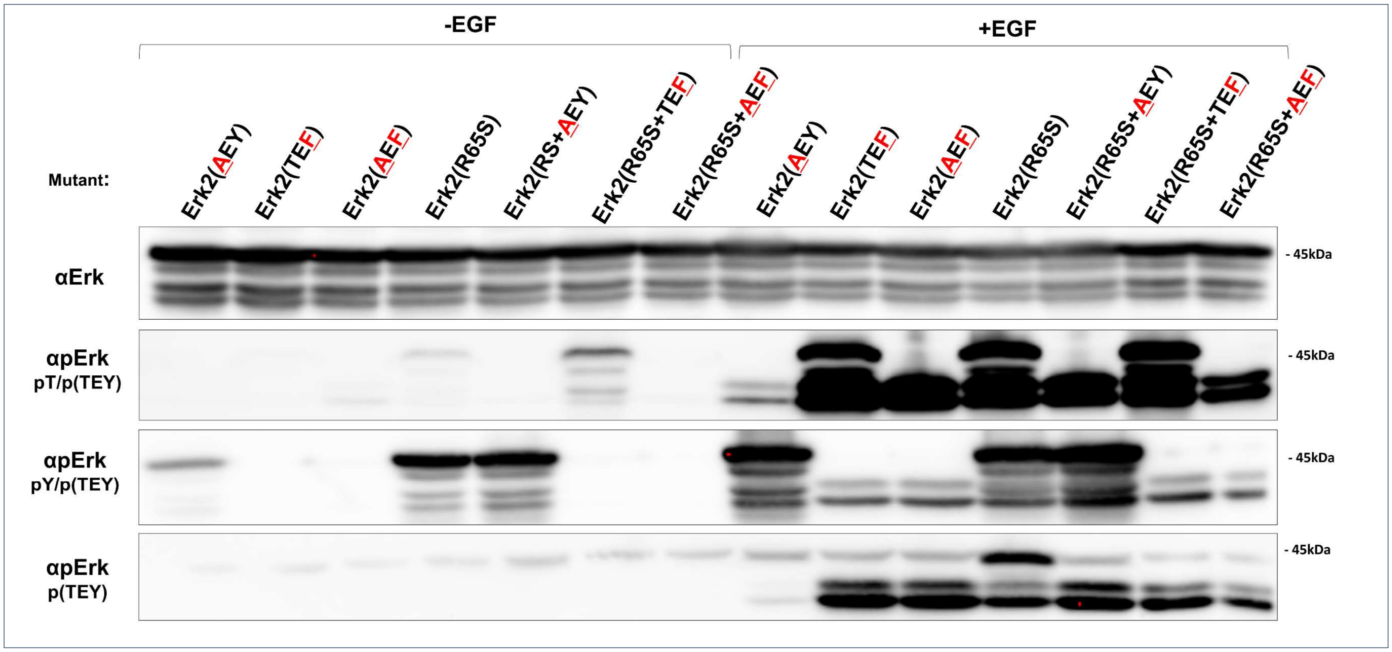

Figure S4. Three different anti-phospho(TEY)-Erk antibodies react with different phosphorylation patterns. To characterize the specificity of the antibodies they were reacted, in a western blot assay, with lysates of HEK293 cells expressing the indicated Erk2 molecules, mutated in the TEY motif.

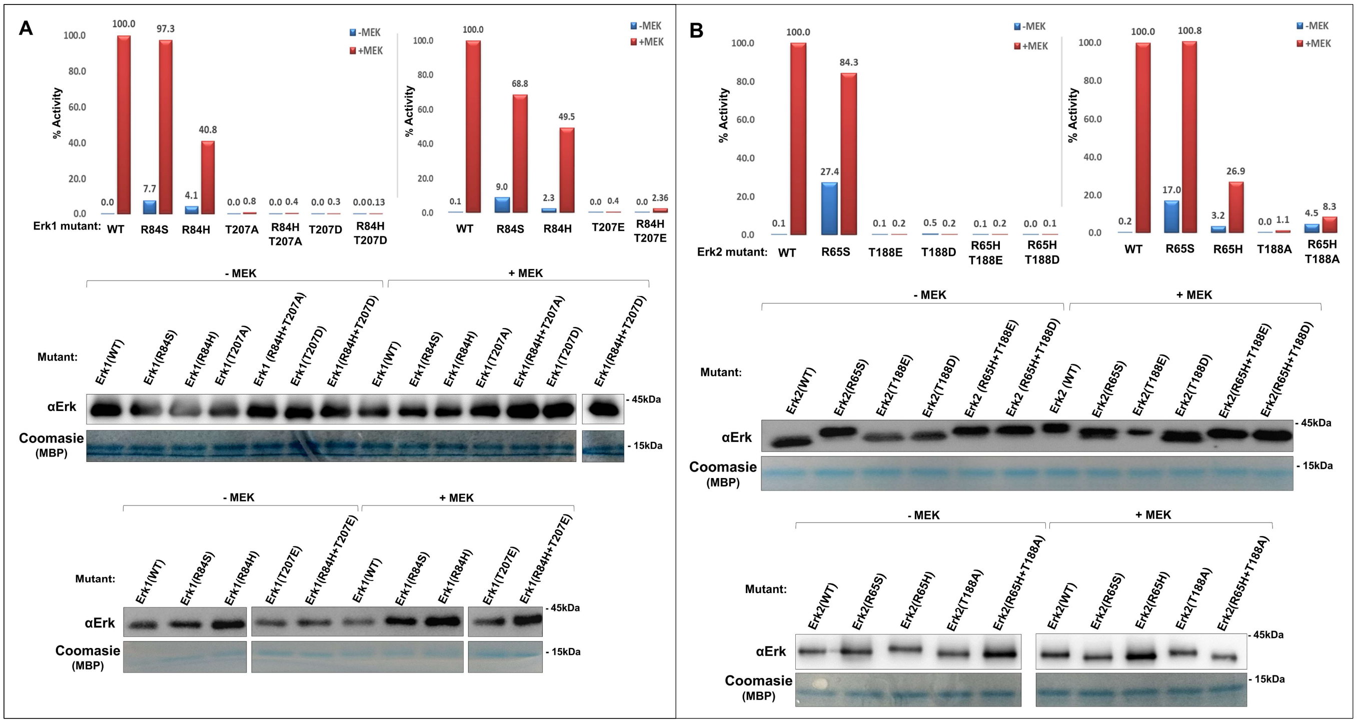

Figure S5. T207/188 is crucial for Erk1/2 catalytic activity. Mutating T207 in $Erk1^{WT}$ or in $Erk1^{R84H}$ (A), $Erk2^{WT}$ or $Erk2^{R65H}$ (B), abolishes both basal and MEK-induced catalytic activity *in vitro*. Catalytic activity of the indicated purified proteins was monitored with or without pre-incubation with active MEK1, using [γ-^{32}p]ATP and MBP as substrates. Reaction mixtures were spotted on filter papers and quantified. Activity of MEK1-activated $Erk1/2^{WT}$ was defined as 100%. In parallel, a sample from each reaction was subjected to a Western blot analysis with antibodies against total Erk1/2 and to SDS-PAGE followed Coomassie blue staining to visualize amounts of MBP substrate. Reactions were performed in triplicates.

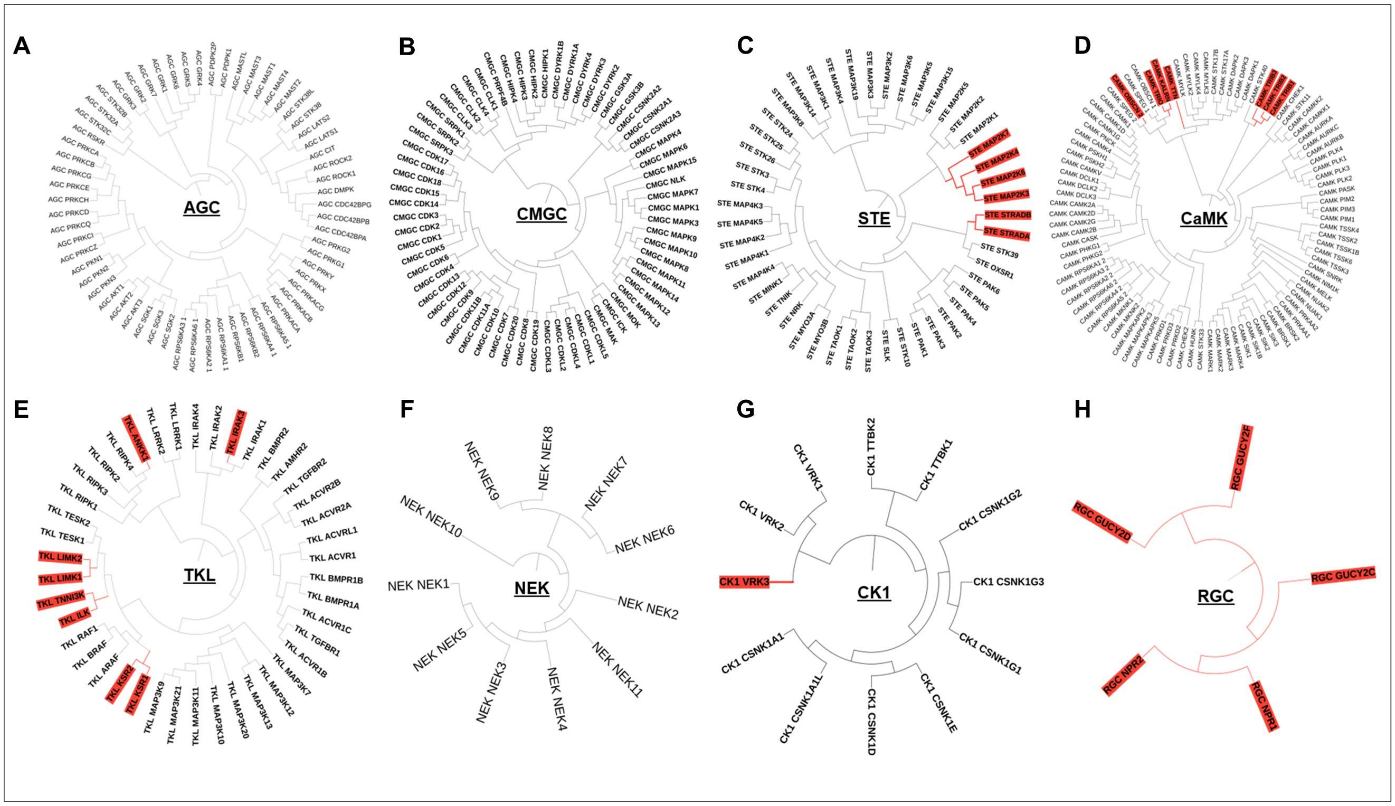

Figure S6. Ser/Thr kinases not possessing T188-orthologs are clustered in particular groups and/or are catalytically inactive. Phylogenic analysis of Ser/Thr kinases shows that in many cases kinases not possessing a T188-ortholog (marked with red) are clustered together in subgroups (e.g. TKL, KSR1/2, LIMK1/2 or STE MAP2K3/4/6/7). Approximately half of the Ser/Thr kinases not possessing T188-orthologs are inactive or are pseudo kinases, namely STRADA/B of the STE family, TRIB1/2/3 of the CaMK family, KSR1/2 and IRAK3 of the TKL family, VRK3 of the CK1 family and all members of RGC group.

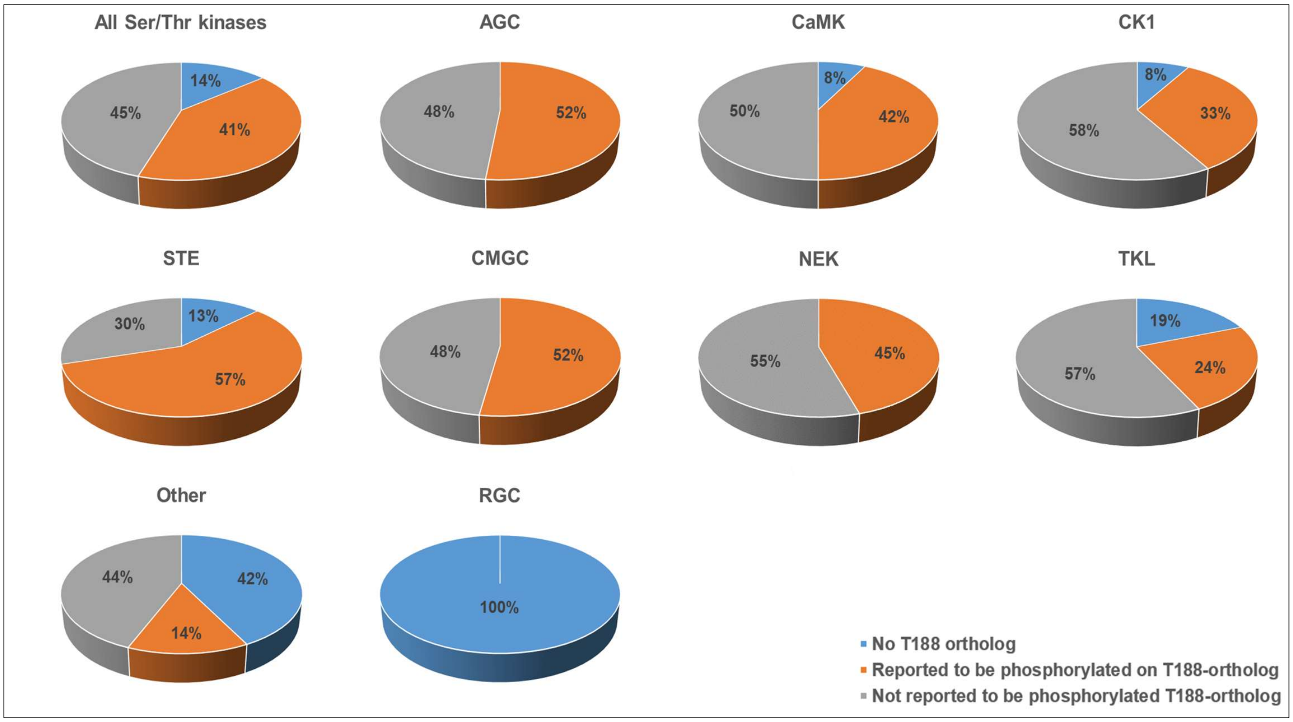

Figure S7. Many EPKs were reported to be phosphorylated on the threonine or serine residues equivalent to Erks' T207/188. Summary of available T188-orthologs phosphoproteomic data (see Table S1) sorted by families. In each EPK sub-family, percentage of kinases not possessing T188 orthologs are indicated in blue, while those found to be phosphorylated on T188-ortholog are indicated in orange and those not reported to be phosphorylated are indicated in grey.

.

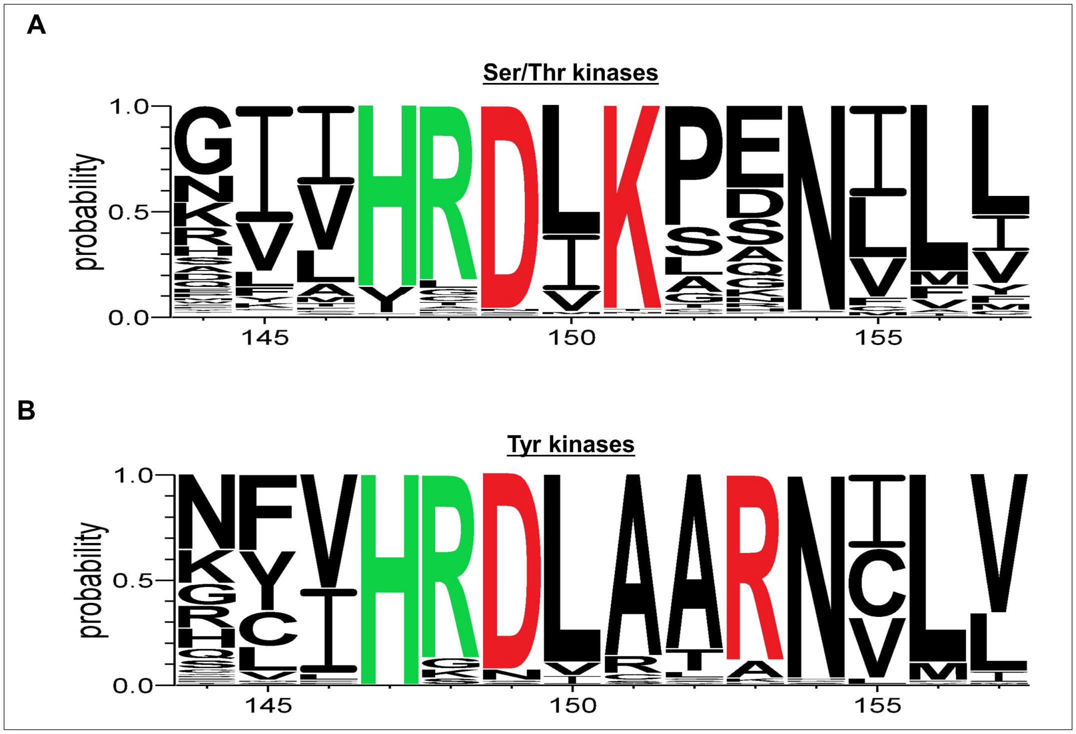

Figure S8. Similarly to T188-orthologs, Lys neighboring HRD motif is invariant in Ser/Thr kinases but absent in Tyrosine kinases. Weblogo representation of multiple sequence alignment of catalytic site with HRD motif from Ser/Thr kinases (A), or Tyrosine kinases and (B). x-axis displays position of amino acid with numbering of PKA and y-axis represents amino acid frequency. Invariant catalytic Asp of HRD motif and neighboring Ser/Thr Lys are marked in red. In Tyrosine kinase family, Lys neighboring HRD in Ser/Thr kinases sequentially and structurally, is substituted by Arg (also marked with red).

Supplementary Table:

Table S1: Summary of available T188-ortholog's phosphoproteomic data in EPKs , 497 kinases.

**Ser/Thr Kinases**					
	**Family**	**Amino acid**	**Position**	**Reported to be phosphorylated**	**Phosphosite link**
**AKT1**	AGC	T	312	No	www.phosphosite.org/proteinAction.action?id=570&showAllSites=true
**AKT2**	AGC	T	313	Yes	www.phosphosite.org/proteinAction.action?id=669&showAllSites=true
**AKT3**	AGC	T	309	Yes	www.phosphosite.org/proteinAction.action?id=670&showAllSites=true
**CDC42BPA**	AGC	T	240	Yes	www.phosphosite.org/proteinAction.action?id=2419&showAllSites=true
**CDC42BPB**	AGC	T	239	No	www.phosphosite.org/proteinAction.action?id=675&showAllSites=true
**CDC42BPG**	AGC	T	234	Yes	www.phosphosite.org/proteinAction.action?id=2417&showAllSites=true
**CIT**	AGC	T	260	No	www.phosphosite.org/proteinAction.action?id=784&showAllSites=true
**DMPK**	AGC	T	234	No	www.phosphosite.org/proteinAction.action?id=691&showAllSites=true
**GRK1**	AGC	T	355	No	www.phosphosite.org/proteinAction.action?id=753&showAllSites=true
**GRK2**	AGC	T	353	No	www.phosphosite.org/proteinAction.action?id=602&showAllSites=true
**GRK3**	AGC	T	353	No	www.phosphosite.org/proteinAction.action?id=2152&showAllSites=true
**GRK4**	AGC	T	349	No	www.phosphosite.org/proteinAction.action?id=698&showAllSites=true
**GRK5**	AGC	T	348	No	www.phosphosite.org/proteinAction.action?id=699&showAllSites=true
**GRK6**	AGC	T	348	No	www.phosphosite.org/proteinAction.action?id=700&showAllSites=true
**GRK7**	AGC	T	353	No	www.phosphosite.org/proteinAction.action?id=2436&showAllSites=true
**LATS1**	AGC	T	913	No	www.phosphosite.org/proteinAction.action?id=2448&showAllSites=true
**LATS2**	AGC	T	876	No	www.phosphosite.org/proteinAction.action?id=705&showAllSites=true
**MAST1**	AGC	T	550	No	www.phosphosite.org/proteinAction.action?id=2441&showAllSites=true
**MAST2**	AGC	T	688	No	www.phosphosite.org/proteinAction.action?id=2439&showAllSites=true
**MAST3**	AGC	T	543	No	www.phosphosite.org/proteinAction.action?id=2438&showAllSites=true
**MAST4**	AGC	T	746	No	www.phosphosite.org/proteinAction.action?id=2444&showAllSites=true
**MASTL**	AGC	T	741	Yes	www.phosphosite.org/proteinAction.action?id=2443&showAllSites=true
**PDPK1**	AGC	T	245	Yes	www.phosphosite.org/proteinAction.action?id=635&showAllSites=true
**PDPK2P**	AGC	T	218	No	www.phosphosite.org/proteinAction.action?id=1872841800&showAllSites=true
**PKN1**	AGC	T	778	Yes	www.phosphosite.org/proteinAction.action?id=650&showAllSites=true
**PKN2**	AGC	T	820	Yes	www.phosphosite.org/proteinAction.action?id=651&showAllSites=true
**PKN3**	AGC	T	722	Yes	www.phosphosite.org/proteinAction.action?id=741&showAllSites=true
**PRKACA**	AGC	T	202	Yes	www.phosphosite.org/proteinAction.action?id=637&showAllSites=true
**PRKACB**	AGC	T	202	Yes	www.phosphosite.org/proteinAction.action?id=743&showAllSites=true

**PRKACG**	AGC	T	202	Yes	www.phosphosite.org/proteinAction.action?id=744&showAllSites=true
**PRKCA**	AGC	T	501	Yes	www.phosphosite.org/proteinAction.action?id=1773&showAllSites=true
**PRKCB**	AGC	T	504	Yes	www.phosphosite.org/proteinAction.action?id=639&showAllSites=true
**PRKCD**	AGC	T	511	Yes	www.phosphosite.org/proteinAction.action?id=882&showAllSites=true
**PRKCE**	AGC	T	570	No	www.phosphosite.org/proteinAction.action?id=1756&showAllSites=true
**PRKCG**	AGC	T	518	Yes	www.phosphosite.org/proteinAction.action?id=643&showAllSites=true
**PRKCH**	AGC	T	517	No	www.phosphosite.org/proteinAction.action?id=642&showAllSites=true
**PRKCI**	AGC	T	416	Yes	www.phosphosite.org/proteinAction.action?id=644&showAllSites=true
**PRKCQ**	AGC	T	542	Yes	www.phosphosite.org/proteinAction.action?id=1760&showAllSites=true
**PRKCZ**	AGC	T	414	No	www.phosphosite.org/proteinAction.action?id=457&showAllSites=true
**PRKG1**	AGC	T	521	Yes	www.phosphosite.org/proteinAction.action?id=745&showAllSites=true
**PRKG2**	AGC	T	613	Yes	www.phosphosite.org/proteinAction.action?id=747&showAllSites=true
**PRKX**	AGC	T	207	No	www.phosphosite.org/proteinAction.action?id=749&showAllSites=true
**PRKY**	AGC	T	207	No	www.phosphosite.org/proteinAction.action?id=2471&showAllSites=true
**ROCK1**	AGC	T	237	No	www.phosphosite.org/proteinAction.action?id=754&showAllSites=true
**ROCK2**	AGC	T	253	Yes	www.phosphosite.org/proteinAction.action?id=755&showAllSites=true
**RPS6KA1**	AGC	T	225	Yes	www.phosphosite.org/proteinAction.action?id=890&showAllSites=true
**RPS6KA2**	AGC	T	222	Yes	www.phosphosite.org/proteinAction.action?id=656&showAllSites=true
**RPS6KA3**	AGC	T	231	Yes	www.phosphosite.org/proteinAction.action?id=655&showAllSites=true
**RPS6KA4**	AGC	T	200	No	www.phosphosite.org/proteinAction.action?id=1779&showAllSites=true
**RPS6KA5**	AGC	T	216	No	www.phosphosite.org/proteinAction.action?id=625&showAllSites=true
**RPS6KA6**	AGC	T	236	Yes	www.phosphosite.org/proteinAction.action?id=2414&showAllSites=true
**RPS6KB1**	AGC	T	256	Yes	www.phosphosite.org/proteinAction.action?id=628&showAllSites=true
**RPS6KB2**	AGC	T	232	No	www.phosphosite.org/proteinAction.action?id=791&showAllSites=true
**RSKR**	AGC	T	266	No	www.phosphosite.org/proteinAction.action?id=2468&showAllSites=true
**SGK1**	AGC	T	260	No	www.phosphosite.org/proteinAction.action?id=660&showAllSites=true
**SGK2**	AGC	T	197	Yes	www.phosphosite.org/proteinAction.action?id=50403036&showAllSites=true
**SGK3**	AGC	T	324	Yes	www.phosphosite.org/proteinAction.action?id=587&showAllSites=true
**STK32A**	AGC	T	183	No	www.phosphosite.org/proteinAction.action?id=10794&showAllSites=true
**STK32B**	AGC	T	184	No	www.phosphosite.org/proteinAction.action?id=2245&showAllSites=true
**STK32C**	AGC	T	253	No	www.phosphosite.org/proteinAction.action?id=2243&showAllSites=true
**STK38**	AGC	T	285	Yes	www.phosphosite.org/proteinAction.action?id=728&showAllSites=true
**STK38L**	AGC	T	286	Yes	www.phosphosite.org/proteinAction.action?id=2450&showAllSites=true
**AURKA**	CAMK	T	292	Yes	www.phosphosite.org/proteinAction.action?id=2173&showAllSites=true
**AURKB**	CAMK	T	236	Yes	www.phosphosite.org/proteinAction.action?id=2170&showAllSites=true
**AURKC**	CAMK	T	202	Yes	https://www.phosphosite.org/proteinAction.action?id=2168&showAllSites=true
**BRSK1**	CAMK	S	193	Yes	www.phosphosite.org/proteinAction.action?id=5125136&showAllSites=true
**BRSK2**	CAMK	S	178	Yes	www.phosphosite.org/proteinAction.action?id=15307&showAllSites=true
**CAMK1**	CAMK	T	181	Yes	www.phosphosite.org/proteinAction.action?id=1430&showAllSites=true
**CAMK1D**	CAMK	T	184	Yes	www.phosphosite.org/proteinAction.action?id=2086&showAllSites=true

**CAMK1G**	CAMK	T	182	No	www.phosphosite.org/proteinAction.action?id=2087&showAllSites=true
**CAMK2A**	CAMK	T	176	No	www.phosphosite.org/proteinAction.action?id=580&showAllSites=true
**CAMK2B**	CAMK	T	177	Yes	www.phosphosite.org/proteinAction.action?id=674&showAllSites=true
**CAMK2D**	CAMK	T	177	No	www.phosphosite.org/proteinAction.action?id=2093&showAllSites=true
**CAMK2G**	CAMK	T	177	No	www.phosphosite.org/proteinAction.action?id=2090&showAllSites=true
**CAMK4**	CAMK	T	204	Yes	www.phosphosite.org/proteinAction.action?id=889&showAllSites=true
**CAMKK1**	CAMK	T	313	No	www.phosphosite.org/proteinAction.action?id=2182&showAllSites=true
**CAMKK2**	CAMK	T	350	Yes	www.phosphosite.org/proteinAction.action?id=2180&showAllSites=true
**CAMKV**	CAMK	T	183	No	www.phosphosite.org/proteinAction.action?id=2107&showAllSites=true
**CASK**	CAMK	T	182	Yes	www.phosphosite.org/proteinAction.action?id=819&showAllSites=true
**CHEK1**	CAMK	T	170	No	www.phosphosite.org/proteinAction.action?id=586&showAllSites=true
**CHEK2**	CAMK	T	387	Yes	www.phosphosite.org/proteinAction.action?id=468&showAllSites=true
**DAPK1**	CAMK	T	180	No	www.phosphosite.org/proteinAction.action?id=590&showAllSites=true
**DAPK2**	CAMK	T	190	No	www.phosphosite.org/proteinAction.action?id=689&showAllSites=true
**DAPK3**	CAMK	T	180	Yes	www.phosphosite.org/proteinAction.action?id=2083&showAllSites=true
**DCLK1**	CAMK	T	550	No	www.phosphosite.org/proteinAction.action?id=811&showAllSites=true
**DCLK2**	CAMK	T	554	No	www.phosphosite.org/proteinAction.action?id=2111&showAllSites=true
**DCLK3**	CAMK	T	516	No	www.phosphosite.org/proteinAction.action?id=2110&showAllSites=true
**HUNK**	CAMK	S	226	No	www.phosphosite.org/proteinAction.action?id=706&showAllSites=true
**KALRN**	CAMK	N			www.phosphosite.org/proteinAction.action?id=778&showAllSites=true
**MAPKAPK2**	CAMK	T	226	Yes	www.phosphosite.org/proteinAction.action?id=716&showAllSites=true
**MAPKAPK3**	CAMK	T	205	Yes	www.phosphosite.org/proteinAction.action?id=717&showAllSites=true
**MAPKAPK5**	CAMK	T	186	Yes	www.phosphosite.org/proteinAction.action?id=649&showAllSites=true
**MARK1**	CAMK	S	219	Yes	www.phosphosite.org/proteinAction.action?id=2100&showAllSites=true
**MARK2**	CAMK	S	212	Yes	www.phosphosite.org/proteinAction.action?id=2099&showAllSites=true
**MARK3**	CAMK	S	215	Yes	www.phosphosite.org/proteinAction.action?id=721&showAllSites=true
**MARK4**	CAMK	S	218	Yes	www.phosphosite.org/proteinAction.action?id=823&showAllSites=true
**MELK**	CAMK	S	171	Yes	www.phosphosite.org/proteinAction.action?id=2101&showAllSites=true
**MKNK1**	CAMK	S	259	No	www.phosphosite.org/proteinAction.action?id=624&showAllSites=true
**MKNK2**	CAMK	S	253	No	www.phosphosite.org/proteinAction.action?id=725&showAllSites=true
**MYLK**	CAMK	T	1624	Yes	www.phosphosite.org/proteinAction.action?id=2112&showAllSites=true
**MYLK2**	CAMK	T	445	Yes	www.phosphosite.org/proteinAction.action?id=2115&showAllSites=true
**MYLK3**	CAMK	T	675	No	www.phosphosite.org/proteinAction.action?id=2114&showAllSites=true
**MYLK4**	CAMK	T	266	No	www.phosphosite.org/proteinAction.action?id=10773&showAllSites=true
**NIM1K**	CAMK	S	233	No	www.phosphosite.org/proteinAction.action?id=3922&showAllSites=true
**NUAK1**	CAMK	S	215	No	www.phosphosite.org/proteinAction.action?id=802&showAllSites=true
**NUAK2**	CAMK	S	212	No	www.phosphosite.org/proteinAction.action?id=2102&showAllSites=true
**OBSCN_1**	CAMK	S	6626	No	www.phosphosite.org/proteinAction.action?id=2129&showAllSites=true
**OBSCN_2**	CAMK	Y			
**PASK**	CAMK	T	1165	Yes	www.phosphosite.org/proteinAction.action?id=2103&showAllSites=true

**PHKG1**	CAMK	T	187	No	www.phosphosite.org/proteinAction.action?id=797&showAllSites=true
**PHKG2**	CAMK	T	190	Yes	www.phosphosite.org/proteinAction.action?id=2116&showAllSites=true
**PIM1**	CAMK	T	204	No	www.phosphosite.org/proteinAction.action?id=2119&showAllSites=true
**PIM2**	CAMK	T	200	No	www.phosphosite.org/proteinAction.action?id=737&showAllSites=true
**PIM3**	CAMK	T	207	No	www.phosphosite.org/proteinAction.action?id=6656&showAllSites=true
**PLK1**	CAMK	T	214	Yes	www.phosphosite.org/proteinAction.action?id=648&showAllSites=true
**PLK2**	CAMK	T	243	No	www.phosphosite.org/proteinAction.action?id=815&showAllSites=true
**PLK3**	CAMK	T	223	No	www.phosphosite.org/proteinAction.action?id=696&showAllSites=true
**PLK4**	CAMK	T	174	Yes	www.phosphosite.org/proteinAction.action?id=765&showAllSites=true
**PLK5**	CAMK	--			www.phosphosite.org/proteinAction.action?id=2821375&showAllSites=true
**PNCK**	CAMK	T	175	Yes	www.phosphosite.org/proteinAction.action?id=14415&showAllSites=true
**PRKAA1**	CAMK	S	187	Yes	www.phosphosite.org/proteinAction.action?id=742&showAllSites=true
**PRKAA2**	CAMK	S	176	Yes	www.phosphosite.org/proteinAction.action?id=572&showAllSites=true
**PRKD1**	CAMK	T	746	Yes	www.phosphosite.org/proteinAction.action?id=1763&showAllSites=true
**PRKD2**	CAMK	T	714	Yes	www.phosphosite.org/proteinAction.action?id=2122&showAllSites=true
**PRKD3**	CAMK	T	739	Yes	www.phosphosite.org/proteinAction.action?id=739&showAllSites=true
**PSKH1**	CAMK	T	260	No	www.phosphosite.org/proteinAction.action?id=790&showAllSites=true
**PSKH2**	CAMK	T	225	No	www.phosphosite.org/proteinAction.action?id=2123&showAllSites=true
**RPS6KA1**	CAMK	T	557	Yes	www.phosphosite.org/proteinAction.action?id=890&showAllSites=true
**RPS6KA2**	CAMK	T	574	Yes	www.phosphosite.org/proteinAction.action?id=656&showAllSites=true
**RPS6KA3**	CAMK	T	581	Yes	www.phosphosite.org/proteinAction.action?id=655&showAllSites=true
**RPS6KA4**	CAMK	T	572	No	www.phosphosite.org/proteinAction.action?id=1779&showAllSites=true
**RPS6KA5**	CAMK	T	585	No	www.phosphosite.org/proteinAction.action?id=625&showAllSites=true
**RPS6KA6**	CAMK	T	585	No	www.phosphosite.org/proteinAction.action?id=2414&showAllSites=true
**SIK1**	CAMK	S	186	Yes	www.phosphosite.org/proteinAction.action?id=3764&showAllSites=true
**SIK1B**	CAMK	S	186	No	www.phosphosite.org/proteinAction.action?id=676507006&showAllSites=true
**SIK2**	CAMK	S	179	Yes	www.phosphosite.org/proteinAction.action?id=2105&showAllSites=true
**SIK3**	CAMK	S	225	Yes	www.phosphosite.org/proteinAction.action?id=22831400&showAllSites=true
**SNRK**	CAMK	S	177	No	www.phosphosite.org/proteinAction.action?id=2106&showAllSites=true
**SPEG_1**	CAMK	T	1760	No	www.phosphosite.org/proteinAction.action?id=2127&showAllSites=true
**SPEG_2**	CAMK	T	3124	No	
**STK11**	CAMK	S	216	No	www.phosphosite.org/proteinAction.action?id=617&showAllSites=true
**STK17A**	CAMK	T	226	No	www.phosphosite.org/proteinAction.action?id=763&showAllSites=true
**STK17B**	CAMK	T	198	No	www.phosphosite.org/proteinAction.action?id=764&showAllSites=true
**STK33**	CAMK	T	286	No	www.phosphosite.org/proteinAction.action?id=2108&showAllSites=true
**STK40**	CAMK	S	236	No	www.phosphosite.org/proteinAction.action?id=2109&showAllSites=true
**TRIB1**	CAMK	C			www.phosphosite.org/proteinAction.action?id=2124&showAllSites=true
**TRIB2**	CAMK	C			www.phosphosite.org/proteinAction.action?id=2125&showAllSites=true
**TRIB3**	CAMK	C			www.phosphosite.org/proteinAction.action?id=2126&showAllSites=true
**TRIO**	CAMK	N			www.phosphosite.org/proteinAction.action?id=2026&showAllSites=true

**TSSK1B**	CAMK	S	178	No	www.phosphosite.org/proteinAction.action?id=787&showAllSites=true
**TSSK2**	CAMK	S	178	No	www.phosphosite.org/proteinAction.action?id=4670&showAllSites=true
**TSSK3**	CAMK	S	172	No	www.phosphosite.org/proteinAction.action?id=2027&showAllSites=true
**TSSK4**	CAMK	S	201	No	www.phosphosite.org/proteinAction.action?id=8250&showAllSites=true
**TSSK6**	CAMK	S	174	No	www.phosphosite.org/proteinAction.action?id=792&showAllSites=true
**TTN**	CAMK	**A**			www.phosphosite.org/proteinAction.action?id=2059&showAllSites=true
**CSNK1A1**	CK1	T	184	Yes	www.phosphosite.org/proteinAction.action?id=688&showAllSites=true
**SNK1A1L**	CK1	T	184	Yes	www.phosphosite.org/proteinAction.action?id=3753&showAllSites=true
**CSNK1D**	CK1	T	176	Yes	www.phosphosite.org/proteinAction.action?id=683&showAllSites=true
**CSNK1E**	CK1	T	176	No	www.phosphosite.org/proteinAction.action?id=684&showAllSites=true
**CSNK1G1**	CK1	T	214	No	www.phosphosite.org/proteinAction.action?id=685&showAllSites=true
**CSNK1G2**	CK1	T	215	Yes	www.phosphosite.org/proteinAction.action?id=686&showAllSites=true
**CSNK1G3**	CK1	T	212	No	www.phosphosite.org/proteinAction.action?id=3751&showAllSites=true
**TTBK1**	CK1	T	202	No	www.phosphosite.org/proteinAction.action?id=3759&showAllSites=true
**TTBK2**	CK1	T	189	No	www.phosphosite.org/proteinAction.action?id=18831310&showAllSites=true
**VRK1**	CK1	T	224	No	www.phosphosite.org/proteinAction.action?id=813&showAllSites=true
**VRK2**	CK1	T	213	No	www.phosphosite.org/proteinAction.action?id=801&showAllSites=true
**VRK3**	CK1	**D**			www.phosphosite.org/proteinAction.action?id=3754&showAllSites=true
**CDK1**	CMGC	T	166	No	www.phosphosite.org/proteinAction.action?id=582&showAllSites=true
**CDK10**	CMGC	T	210	No	www.phosphosite.org/proteinAction.action?id=677&showAllSites=true
**CDK11A**	CMGC	T	588	Yes	www.phosphosite.org/proteinAction.action?id=23582&showAllSites=true
**CDK11B**	CMGC	T	600	Yes	www.phosphosite.org/proteinAction.action?id=2134&showAllSites=true
**CDK12**	CMGC	T	898	No	www.phosphosite.org/proteinAction.action?id=2133&showAllSites=true
**CDK13**	CMGC	T	876	No	www.phosphosite.org/proteinAction.action?id=2132&showAllSites=true
**CDK14**	CMGC	T	294	No	www.phosphosite.org/proteinAction.action?id=736&showAllSites=true
**CDK15**	CMGC	T	262	No	www.phosphosite.org/proteinAction.action?id=3760&showAllSites=true
**CDK16**	CMGC	T	324	No	www.phosphosite.org/proteinAction.action?id=734&showAllSites=true
**CDK17**	CMGC	T	351	No	www.phosphosite.org/proteinAction.action?id=735&showAllSites=true
**CDK18**	CMGC	T	303	No	www.phosphosite.org/proteinAction.action?id=2135&showAllSites=true
**CDK19**	CMGC	T	196	No	www.phosphosite.org/proteinAction.action?id=2130&showAllSites=true
**CDK2**	CMGC	T	165	Yes	www.phosphosite.org/proteinAction.action?id=583&showAllSites=true
**CDK20**	CMGC	T	166	No	www.phosphosite.org/proteinAction.action?id=676&showAllSites=true
**CDK3**	CMGC	T	165	No	www.phosphosite.org/proteinAction.action?id=678&showAllSites=true
**CDK4**	CMGC	T	177	No	www.phosphosite.org/proteinAction.action?id=584&showAllSites=true
**CDK5**	CMGC	T	164	No	www.phosphosite.org/proteinAction.action?id=585&showAllSites=true
**CDK6**	CMGC	T	182	No	www.phosphosite.org/proteinAction.action?id=679&showAllSites=true
**CDK7**	CMGC	T	175	Yes	www.phosphosite.org/proteinAction.action?id=578&showAllSites=true
**CDK8**	CMGC	T	196	No	www.phosphosite.org/proteinAction.action?id=680&showAllSites=true
**CDK9**	CMGC	T	191	No	www.phosphosite.org/proteinAction.action?id=681&showAllSites=true
**CDKL1**	CMGC	T	164	No	www.phosphosite.org/proteinAction.action?id=703&showAllSites=true

**CDKL2**	CMGC	T	164	No	www.phosphosite.org/proteinAction.action?id=704&showAllSites=true
**CDKL3**	CMGC	T	163	No	www.phosphosite.org/proteinAction.action?id=805&showAllSites=true
**CDKL4**	CMGC	T	163	No	www.phosphosite.org/proteinAction.action?id=7384500&showAllSites=true
**CDKL5**	CMGC	T	174	Yes	www.phosphosite.org/proteinAction.action?id=772&showAllSites=true
**CLK1**	CMGC	T	342	Yes	www.phosphosite.org/proteinAction.action?id=2136&showAllSites=true
**CLK2**	CMGC	T	344	Yes	www.phosphosite.org/proteinAction.action?id=810&showAllSites=true
**CLK3**	CMGC	T	485	No	www.phosphosite.org/proteinAction.action?id=2137&showAllSites=true
**CLK4**	CMGC	T	340	Yes	www.phosphosite.org/proteinAction.action?id=682&showAllSites=true
**CSNK2A1**	CMGC	S	194	Yes	www.phosphosite.org/proteinAction.action?id=798&showAllSites=true
**CSNK2A2**	CMGC	S	195	Yes	www.phosphosite.org/proteinAction.action?id=687&showAllSites=true
**CSNK2A3**	CMGC	S	194	No	www.phosphosite.org/proteinAction.action?id=27779302&showAllSites=true
**DYRK1A**	CMGC	S	324	Yes	www.phosphosite.org/proteinAction.action?id=692&showAllSites=true
**DYRK1B**	CMGC	S	276	Yes	www.phosphosite.org/proteinAction.action?id=692&showAllSites=true
**DYRK2**	CMGC	S	385	No	www.phosphosite.org/proteinAction.action?id=693&showAllSites=true
**DYRK3**	CMGC	S	327	Yes	www.phosphosite.org/proteinAction.action?id=694&showAllSites=true
**DYRK4**	CMGC	S	267	No	www.phosphosite.org/proteinAction.action?id=695&showAllSites=true
**GSK3A**	CMGC	S	282	Yes	www.phosphosite.org/proteinAction.action?id=603&showAllSites=true
**GSK3B**	CMGC	S	219	Yes	www.phosphosite.org/proteinAction.action?id=604&showAllSites=true
**HIPK1**	CMGC	S	355	Yes	www.phosphosite.org/proteinAction.action?id=4227&showAllSites=true
**HIPK2**	CMGC	S	364	Yes	www.phosphosite.org/proteinAction.action?id=701&showAllSites=true
**HIPK3**	CMGC	S	362	Yes	www.phosphosite.org/proteinAction.action?id=702&showAllSites=true
**HIPK4**	CMGC	S	178	No	www.phosphosite.org/proteinAction.action?id=3761&showAllSites=true
**ICK**	CMGC	T	162	Yes	www.phosphosite.org/proteinAction.action?id=2139&showAllSites=true
**MAK**	CMGC	T	162	Yes	www.phosphosite.org/proteinAction.action?id=812&showAllSites=true
**MAPK1**	CMGC	T	190	Yes	www.phosphosite.org/proteinAction.action?id=832&showAllSites=true
**MAPK10**	CMGC	T	226	Yes	www.phosphosite.org/proteinAction.action?id=713&showAllSites=true
**MAPK11**	CMGC	T	185	Yes	www.phosphosite.org/proteinAction.action?id=627&showAllSites=true
**MAPK12**	CMGC	T	188	No	www.phosphosite.org/proteinAction.action?id=658&showAllSites=true
**MAPK13**	CMGC	T	185	Yes	www.phosphosite.org/proteinAction.action?id=714&showAllSites=true
**MAPK14**	CMGC	T	185	Yes	www.phosphosite.org/proteinAction.action?id=626&showAllSites=true
**MAPK15**	CMGC	T	180	No	www.phosphosite.org/proteinAction.action?id=2138&showAllSites=true
**MAPK3**	CMGC	T	207	Yes	www.phosphosite.org/proteinAction.action?id=595&showAllSites=true
**MAPK4**	CMGC	T	191	No	www.phosphosite.org/proteinAction.action?id=715&showAllSites=true
**MAPK6**	CMGC	T	194	No	www.phosphosite.org/proteinAction.action?id=596&showAllSites=true
**MAPK7**	CMGC	T	224	Yes	www.phosphosite.org/proteinAction.action?id=7570&showAllSites=true
**MAPK8**	CMGC	T	188	Yes	www.phosphosite.org/proteinAction.action?id=613&showAllSites=true
**MAPK9**	CMGC	T	188	Yes	www.phosphosite.org/proteinAction.action?id=1234&showAllSites=true
**MOK**	CMGC	T	164	No	www.phosphosite.org/proteinAction.action?id=726&showAllSites=true
**NLK**	CMGC	T	303	Yes	www.phosphosite.org/proteinAction.action?id=809&showAllSites=true
**PRPF4B**	CMGC	S	852	Yes	www.phosphosite.org/proteinAction.action?id=817&showAllSites=true

**SRPK1**	CMGC	T	514	Yes	www.phosphosite.org/proteinAction.action?id=6658&showAllSites=true
**SRPK2**	CMGC	T	547	Yes	www.phosphosite.org/proteinAction.action?id=760&showAllSites=true
**SRPK3**	CMGC	T	462	Yes	www.phosphosite.org/proteinAction.action?id=767&showAllSites=true
**NEK1**	NEK	T	166	No	www.phosphosite.org/proteinAction.action?id=2193&showAllSites=true
**NEK10**	NEK	T	692	No	www.phosphosite.org/proteinAction.action?id=5021375&showAllSites=true
**NEK11**	NEK	T	195	No	www.phosphosite.org/proteinAction.action?id=2198&showAllSites=true
**NEK2**	NEK	T	179	Yes	www.phosphosite.org/proteinAction.action?id=729&showAllSites=true
**NEK3**	NEK	T	165	Yes	www.phosphosite.org/proteinAction.action?id=730&showAllSites=true
**NEK4**	NEK	T	169	No	www.phosphosite.org/proteinAction.action?id=766&showAllSites=true
**NEK5**	NEK	T	167	No	www.phosphosite.org/proteinAction.action?id=18823&showAllSites=true
**NEK6**	NEK	T	210	Yes	www.phosphosite.org/proteinAction.action?id=731&showAllSites=true
**NEK7**	NEK	T	199	Yes	www.phosphosite.org/proteinAction.action?id=1752&showAllSites=true
**NEK8**	NEK	T	166	No	www.phosphosite.org/proteinAction.action?id=4718&showAllSites=true
**NEK9**	NEK	T	214	Yes	www.phosphosite.org/proteinAction.action?id=2195&showAllSites=true
**AAK1**	OTHER	T	222	Yes	www.phosphosite.org/proteinAction.action?id=2190&showAllSites=true
**BMP2K**	OTHER	T	226	No	www.phosphosite.org/proteinAction.action?id=2191&showAllSites=true
**BUB1**	OTHER	T	968	No	www.phosphosite.org/proteinAction.action?id=673&showAllSites=true
**BUB1B**	OTHER	**V**			www.phosphosite.org/proteinAction.action?id=2176&showAllSites=true
**CDC7**	OTHER	T	376	Yes	www.phosphosite.org/proteinAction.action?id=806&showAllSites=true
**CHUK**	OTHER	T	184	No	www.phosphosite.org/proteinAction.action?id=461&showAllSites=true
**DSTYK**	OTHER	T	812	No	www.phosphosite.org/proteinAction.action?id=2214&showAllSites=true
**EIF2AK1**	OTHER	T	493	Yes	www.phosphosite.org/proteinAction.action?id=1674&showAllSites=true
**EIF2AK2**	OTHER	T	451	Yes	www.phosphosite.org/proteinAction.action?id=646&showAllSites=true
**EIF2AK3**	OTHER	T	987	No	www.phosphosite.org/proteinAction.action?id=636&showAllSites=true
**EIF2AK4_1**	OTHER	T	462	No	https://www.phosphosite.org/proteinAction.action?id=1658&showAllSites=true
**EIF2AK4_2**	OTHER	T	904	No	
**ERN1**	OTHER	T	734	No	
**ERN2**	OTHER	T	683	No	www.phosphosite.org/proteinAction.action?id=1304679&showAllSites=true
**GAK**	OTHER	T	222	No	www.phosphosite.org/proteinAction.action?id=2187&showAllSites=true
**HASPIN**	OTHER	**Q**			www.phosphosite.org/proteinAction.action?id=2185&showAllSites=true
**IKBKB**	OTHER	T	185	No	www.phosphosite.org/proteinAction.action?id=459&showAllSites=true
**IKBKE**	OTHER	T	176	No	www.phosphosite.org/proteinAction.action?id=1136&showAllSites=true
**MLKL**	OTHER	S	373	Yes	www.phosphosite.org/proteinAction.action?id=2016&showAllSites=true
**MOS**	OTHER	T	242	No	www.phosphosite.org/proteinAction.action?id=814&showAllSites=true
**NRBP1**	OTHER	**N**			www.phosphosite.org/proteinAction.action?id=2211&showAllSites=true
**NRBP12**	OTHER	**N**			
**PAN3**	OTHER	**--**			www.phosphosite.org/proteinAction.action?id=2614317&showAllSites=true
**PBK**	OTHER	T	209	No	www.phosphosite.org/proteinAction.action?id=2235&showAllSites=true
**PDIK1L**	OTHER	T	221	No	www.phosphosite.org/proteinAction.action?id=2205&showAllSites=true
**PEAK1**	OTHER	**D**			www.phosphosite.org/proteinAction.action?id=2204&showAllSites=true

**PEAK3**	OTHER	**--**			www.phosphosite.org/proteinAction.action?id=15251504&showAllSites=true
**PIK3R4**	OTHER	**R**			www.phosphosite.org/proteinAction.action?id=2239&showAllSites=true
**PINK1**	OTHER	**N**			www.phosphosite.org/proteinAction.action?id=2201&showAllSites=true
**PKDCC**	OTHER	**I**			www.phosphosite.org/proteinAction.action?id=22299&showAllSites=true
**PKMYT1**	OTHER	**D**			www.phosphosite.org/proteinAction.action?id=1820&showAllSites=true
**POMK**	OTHER	**H**			www.phosphosite.org/proteinAction.action?id=2216&showAllSites=true
**PRAG1**	OTHER	S	1235	No	www.phosphosite.org/proteinAction.action?id=3935&showAllSites=true
**PXK**	OTHER	**L**			www.phosphosite.org/proteinAction.action?id=2230&showAllSites=true
**RNASEL**	OTHER	**--**			www.phosphosite.org/proteinAction.action?id=2222&showAllSites=true
**RPS6KC1**	OTHER	**E**			www.phosphosite.org/proteinAction.action?id=2470&showAllSites=true
**RPS6KL1**	OTHER	**D**			www.phosphosite.org/proteinAction.action?id=2469&showAllSites=true
**SBK1**	OTHER	T	211	No	www.phosphosite.org/proteinAction.action?id=1275502&showAllSites=true
**SBK2**	OTHER	**P**			www.phosphosite.org/proteinAction.action?id=7384502&showAllSites=true
**SBK3**	OTHER	**P**			www.phosphosite.org/proteinAction.action?id=7384504&showAllSites=true
**SCYL1**	OTHER	**L**			www.phosphosite.org/proteinAction.action?id=2224&showAllSites=true
**SCYL2**	OTHER	**N**			www.phosphosite.org/proteinAction.action?id=2228&showAllSites=true
**SCYL3**	OTHER	**D**			www.phosphosite.org/proteinAction.action?id=2226&showAllSites=true
**STK16**	OTHER	T	195	No	www.phosphosite.org/proteinAction.action?id=762&showAllSites=true
**STK31**	OTHER	**D**			www.phosphosite.org/proteinAction.action?id=2217&showAllSites=true
**STK35**	OTHER	S	418	Yes	www.phosphosite.org/proteinAction.action?id=2207&showAllSites=true
**STK36**	OTHER	T	163	No	www.phosphosite.org/proteinAction.action?id=697&showAllSites=true
**STKLD1**	OTHER	**R**			www.phosphosite.org/proteinAction.action?id=15189&showAllSites=true
**TBCK**	OTHER	**Y**			www.phosphosite.org/proteinAction.action?id=2231&showAllSites=true
**TBK1**	OTHER	**T**	176	No	www.phosphosite.org/proteinAction.action?id=825&showAllSites=true
**TEX14**	OTHER	**L**			www.phosphosite.org/proteinAction.action?id=2209&showAllSites=true
**TLK1**	OTHER	**T**	633	No	www.phosphosite.org/proteinAction.action?id=775&showAllSites=true
**TLK2**	OTHER	**T**	640	No	www.phosphosite.org/proteinAction.action?id=776&showAllSites=true
**TP53RK**	OTHER	**--**			www.phosphosite.org/proteinAction.action?id=2178&showAllSites=true
**TTK(Mps1)**	OTHER	**T**	686	Yes	www.phosphosite.org/proteinAction.action?id=779&showAllSites=true
**UHMK1**	OTHER	**T**	194	No	www.phosphosite.org/proteinAction.action?id=2219&showAllSites=true
**ULK1**	OTHER	**S**	184	No	www.phosphosite.org/proteinAction.action?id=796&showAllSites=true
**ULK2**	OTHER	**S**	177	No	www.phosphosite.org/proteinAction.action?id=807&showAllSites=true
**ULK3**	OTHER	**S**	176	Yes	www.phosphosite.org/proteinAction.action?id=2812801&showAllSites=true
**ULK4**	OTHER	**S**	182	No	www.phosphosite.org/proteinAction.action?id=2237&showAllSites=true
**WEE1**	OTHER	**D**	479		www.phosphosite.org/proteinAction.action?id=821&showAllSites=true
**WEE2**	OTHER	**D**	396		www.phosphosite.org/proteinAction.action?id=2812805&showAllSites=true
**WNK1**	OTHER	T	386	No	www.phosphosite.org/proteinAction.action?id=748&showAllSites=true
**WNK2**	OTHER	T	360	No	www.phosphosite.org/proteinAction.action?id=2242&showAllSites=true
**WNK3**	OTHER	T	312	No	www.phosphosite.org/proteinAction.action?id=803&showAllSites=true
**WNK4**	OTHER	T	339	No	www.phosphosite.org/proteinAction.action?id=6663&showAllSites=true

**RGC_GUCY2C**	RGC	K			www.phosphosite.org/proteinAction.action?id=2374&showAllSites=true
**RGC_GUCY2D**	RGC	D			www.phosphosite.org/proteinAction.action?id=2370&showAllSites=true
**RGC_GUCY2F**	RGC	E			www.phosphosite.org/proteinAction.action?id=2372&showAllSites=true
**RGC_NPR1**	RGC	K			www.phosphosite.org/proteinAction.action?id=916&showAllSites=true
**RGC_NPR2**	RGC	K			www.phosphosite.org/proteinAction.action?id=1079&showAllSites=true
**MAP2K1**	STE	T	224	No	www.phosphosite.org/proteinAction.action?id=619&showAllSites=true
**MAP2K2**	STE	T	230	No	www.phosphosite.org/proteinAction.action?id=719&showAllSites=true
**MAP2K3**	STE	C			www.phosphosite.org/proteinAction.action?id=621&showAllSites=true
**MAP2K4**	STE	C			www.phosphosite.org/proteinAction.action?id=659&showAllSites=true
**MAP2K5**	STE	T	319	No	www.phosphosite.org/proteinAction.action?id=720&showAllSites=true
**MAP2K6**	STE	C			www.phosphosite.org/proteinAction.action?id=894&showAllSites=true
**MAP2K7**	STE	C			www.phosphosite.org/proteinAction.action?id=1002&showAllSites=true
**MAP3K1**	STE	T	1412	Yes	www.phosphosite.org/proteinAction.action?id=1736&showAllSites=true
**MAP3K14**	STE	T	559	Yes	www.phosphosite.org/proteinAction.action?id=464&showAllSites=true
**MAP3K15**	STE	T	810	No	www.phosphosite.org/proteinAction.action?id=18239&showAllSites=true
**MAP3K19**	STE	T	1230	No	www.phosphosite.org/proteinAction.action?id=1911504&showAllSites=true
**MAP3K2**	STE	T	524	Yes	www.phosphosite.org/proteinAction.action?id=841&showAllSites=true
**MAP3K3**	STE	T	530	Yes	www.phosphosite.org/proteinAction.action?id=842&showAllSites=true
**MAP3K4**	STE	T	1505	Yes	www.phosphosite.org/proteinAction.action?id=840&showAllSites=true
**MAP3K5**	STE	T	842	Yes	www.phosphosite.org/proteinAction.action?id=575&showAllSites=true
**MAP3K6**	STE	T	810	No	www.phosphosite.org/proteinAction.action?id=710&showAllSites=true
**MAP3K8**	STE	T	290	Yes	www.phosphosite.org/siteAction.action?id=20951
**MAP4K1**	STE	T	175	Yes	www.phosphosite.org/proteinAction.action?id=1180&showAllSites=true
**MAP4K2**	STE	T	174	Yes	www.phosphosite.org/proteinAction.action?id=2489&showAllSites=true
**MAP4K3**	STE	T	174	No	www.phosphosite.org/proteinAction.action?id=2492&showAllSites=true
**MAP4K4**	STE	T	191	Yes	www.phosphosite.org/proteinAction.action?id=2496&showAllSites=true
**MAP4K5**	STE	T	178	Yes	www.phosphosite.org/proteinAction.action?id=712&showAllSites=true
**MINK1**	STE	T	191	Yes	www.phosphosite.org/proteinAction.action?id=723&showAllSites=true
**MYO3A**	STE	T	188	Yes	www.phosphosite.org/proteinAction.action?id=822&showAllSites=true
**MYO3B**	STE	T	194	No	www.phosphosite.org/proteinAction.action?id=2511&showAllSites=true
**NRK**	STE	T	215	No	www.phosphosite.org/proteinAction.action?id=3921&showAllSites=true
**OXSR1**	STE	T	189	Yes	www.phosphosite.org/proteinAction.action?id=824&showAllSites=true
**PAK1**	STE	T	427	Yes	www.phosphosite.org/proteinAction.action?id=630&showAllSites=true
**PAK2**	STE	T	406	Yes	www.phosphosite.org/proteinAction.action?id=631&showAllSites=true
**PAK3**	STE	T	440	Yes	www.phosphosite.org/proteinAction.action?id=632&showAllSites=true
**PAK4**	STE	T	478	Yes	www.phosphosite.org/proteinAction.action?id=633&showAllSites=true
**PAK5**	STE	T	606	Yes	www.phosphosite.org/proteinAction.action?id=732&showAllSites=true
**PAK6**	STE	T	564	Yes	www.phosphosite.org/proteinAction.action?id=733&showAllSites=true
**SLK**	STE	T	193	Yes	www.phosphosite.org/proteinAction.action?id=2519&showAllSites=true
**STK10**	STE	T	195	Yes	www.phosphosite.org/proteinAction.action?id=761&showAllSites=true

**STK24**	STE	T	194	Yes	www.phosphosite.org/proteinAction.action?id=768&showAllSites=true
**STK25**	STE	T	178	Yes	www.phosphosite.org/proteinAction.action?id=769&showAllSites=true
**STK26**	STE	T	182	Yes	www.phosphosite.org/proteinAction.action?id=2509&showAllSites=true
**STK3**	STE	T	184	No	www.phosphosite.org/proteinAction.action?id=770&showAllSites=true
**STK39**	STE	T	235	Yes	www.phosphosite.org/proteinAction.action?id=758&showAllSites=true
**STK4**	STE	T	187	No	www.phosphosite.org/proteinAction.action?id=771&showAllSites=true
**STRADA**	STE	V			www.phosphosite.org/proteinAction.action?id=2521&showAllSites=true
**STRADB**	STE	V			www.phosphosite.org/proteinAction.action?id=2522&showAllSites=true
**TAOK1**	STE	T	185	No	www.phosphosite.org/proteinAction.action?id=2525&showAllSites=true
**TAOK2**	STE	T	185	Yes	www.phosphosite.org/proteinAction.action?id=820&showAllSites=true
**TAOK3**	STE	T	181	No	www.phosphosite.org/proteinAction.action?id=2524&showAllSites=true
**TNIK**	STE	T	191	No	www.phosphosite.org/proteinAction.action?id=2498&showAllSites=true
**ACVR1**	TKL	T	378	No	www.phosphosite.org/proteinAction.action?id=12612&showAllSites=true
**ACVR1B**	TKL	T	377	No	www.phosphosite.org/proteinAction.action?id=2010&showAllSites=true
**ACVR1C**	TKL	T	365	No	www.phosphosite.org/proteinAction.action?id=2012&showAllSites=true
**ACVR2A**	TKL	T	362	No	www.phosphosite.org/proteinAction.action?id=782&showAllSites=true
**ACVR2B**	TKL	T	361	No	www.phosphosite.org/proteinAction.action?id=783&showAllSites=true
**ACVRL1**	TKL	T	372	No	www.phosphosite.org/proteinAction.action?id=818&showAllSites=true
**AMHR2**	TKL	T	383	No	www.phosphosite.org/proteinAction.action?id=2013&showAllSites=true
**ANKK1**	TKL	M			www.phosphosite.org/proteinAction.action?id=2002&showAllSites=true
**ARAF**	TKL	S	469	No	www.phosphosite.org/proteinAction.action?id=671&showAllSites=true
**BMPR1A**	TKL	T	403	No	www.phosphosite.org/proteinAction.action?id=2007&showAllSites=true
**BMPR1B**	TKL	T	374	No	www.phosphosite.org/proteinAction.action?id=816&showAllSites=true
**BMPR2**	TKL	T	379	Yes	www.phosphosite.org/proteinAction.action?id=672&showAllSites=true
**BRAF**	TKL	S	616	Yes	www.phosphosite.org/proteinAction.action?id=577&showAllSites=true
**ILK**	TKL	A			www.phosphosite.org/proteinAction.action?id=607&showAllSites=true
**IRAK1**	TKL	T	387	Yes	www.phosphosite.org/proteinAction.action?id=789&showAllSites=true
**IRAK2**	TKL	S	378	No	www.phosphosite.org/proteinAction.action?id=788&showAllSites=true
**IRAK3**	TKL	H			www.phosphosite.org/proteinAction.action?id=1996&showAllSites=true
**IRAK4**	TKL	T	351	No	www.phosphosite.org/proteinAction.action?id=1998&showAllSites=true
**KSR1**	TKL	W			www.phosphosite.org/proteinAction.action?id=2001&showAllSites=true
**KSR2**	TKL	W			www.phosphosite.org/proteinAction.action?id=3796&showAllSites=true
**LIMK1**	TKL	N			www.phosphosite.org/proteinAction.action?id=616&showAllSites=true
**LIMK2**	TKL	N			www.phosphosite.org/proteinAction.action?id=828&showAllSites=true
**LRRK1**	TKL	T	1427	Yes	www.phosphosite.org/proteinAction.action?id=3794&showAllSites=true
**LRRK2**	TKL	T	2035	Yes	www.phosphosite.org/proteinAction.action?id=7124&showAllSites=true
**MAP3K10**	TKL	T	266	No	www.phosphosite.org/proteinAction.action?id=724&showAllSites=true
**MAP3K11**	TKL	T	285	No	www.phosphosite.org/proteinAction.action?id=623&showAllSites=true
**MAP3K12**	TKL	T	273	No	www.phosphosite.org/proteinAction.action?id=708&showAllSites=true
**MAP3K13**	TKL	T	316	No	www.phosphosite.org/proteinAction.action?id=709&showAllSites=true

**MAP3K20**	TKL	T	169	No	www.phosphosite.org/proteinAction.action?id=799&showAllSites=true
**MAP3K21**	TKL	T	307	No	www.phosphosite.org/proteinAction.action?id=2000&showAllSites=true
**MAP3K7**	TKL	S	192	Yes	www.phosphosite.org/proteinAction.action?id=664&showAllSites=true
**MAP3K9**	TKL	T	312	Yes	www.phosphosite.org/proteinAction.action?id=804&showAllSites=true
**RAF1**	TKL	S	508	No	www.phosphosite.org/proteinAction.action?id=653&showAllSites=true
**RIPK1**	TKL	T	189	No	www.phosphosite.org/proteinAction.action?id=750&showAllSites=true
**RIPK2**	TKL	T	189	No	www.phosphosite.org/proteinAction.action?id=751&showAllSites=true
**RIPK3**	TKL	T	182	Yes	www.phosphosite.org/proteinAction.action?id=752&showAllSites=true
**RIPK4**	TKL	T	184	Yes	www.phosphosite.org/proteinAction.action?id=573&showAllSites=true
**TESK1**	TKL	S	220	Yes	www.phosphosite.org/proteinAction.action?id=773&showAllSites=true
**TESK2**	TKL	S	219	No	www.phosphosite.org/proteinAction.action?id=774&showAllSites=true
**TGFBR1**	TKL	T	375	No	www.phosphosite.org/proteinAction.action?id=665&showAllSites=true
**TGFBR2**	TKL	T	421	No	www.phosphosite.org/proteinAction.action?id=666&showAllSites=true
**TNNI3K**	TKL	N			www.phosphosite.org/proteinAction.action?id=3795&showAllSites=true

Tyrosine kinases

	Family	Amino acid
**AATK**	TYR	P
**ABL1**	TYR	P
**ABL2**	TYR	P
**ALK**	TYR	P
**AXL**	TYR	P
**BLK**	TYR	P
**BMX**	TYR	P
**BTK**	TYR	P
**CSF1R**	TYR	P
**CSK**	TYR	P
**DDR1**	TYR	P
**DDR2**	TYR	P
**EGFR**	TYR	P
**EPHA1**	TYR	P
**EPHA10**	TYR	P
**EPHA2**	TYR	P
**EPHA3**	TYR	P
**EPHA4**	TYR	P
**EPHA5**	TYR	P
**EPHA6**	TYR	P
**EPHA7**	TYR	P
**EPHA8**	TYR	P

**EPHB1**	TYR	P
**EPHB2**	TYR	P
**EPHB3**	TYR	P
**EPHB4**	TYR	P
**EPHB6**	TYR	L
**ERBB2**	TYR	P
**ERBB3**	TYR	P
**ERBB4**	TYR	P
**FER**	TYR	P
**FES**	TYR	P
**FGFR1**	TYR	P
**FGFR2**	TYR	P
**FGFR3**	TYR	P
**FGFR4**	TYR	P
**FGR**	TYR	P
**FLT1**	TYR	P
**FLT3**	TYR	P
**FLT4**	TYR	P
**FRK**	TYR	P
**FYN**	TYR	P
**HCK**	TYR	P
**IGF1R**	TYR	P
**INSR**	TYR	P
**INSRR**	TYR	P
**ITK4**	TYR	P
**JAK1_1**	TYR	R
**JAK1_2**	TYR	P
**JAK2_1**	TYR	R
**JAK2_2**	TYR	P
**JAK3_1**	TYR	R
**JAK3_2**	TYR	P
**KDR**	TYR	P
**KIT**	TYR	P
**LCK**	TYR	P
**LMTK2**	TYR	P
**LMTK3**	TYR	P
**LTK**	TYR	P
**LYN**	TYR	P
**MATK**	TYR	P
**MERTK**	TYR	P

**MET**	TYR	P
**MST1R**	TYR	P
**MUSK**	TYR	P
**NTRK1**	TYR	P
**NTRK2**	TYR	P
**NTRK3**	TYR	P
**PDGFRA**	TYR	P
**PDGFRB**	TYR	P
**PTK2**	TYR	P
**PTK2B**	TYR	P
**PTK6**	TYR	P
**PTK7**	TYR	P
**RET**	TYR	P
**ROR1**	TYR	P
**ROR2**	TYR	P
**ROS1**	TYR	P
**RYK**	TYR	P
**SRC**	TYR	P
**SRMS**	TYR	P
**STYK1**	TYR	P
**SYK**	TYR	P
**TEC**	TYR	P
**TEK**	TYR	P
**TIE1**	TYR	P
**TNK1**	TYR	P
**TNK2**	TYR	P
**TXK**	TYR	P
**TYK2_1**	TYR	R
**TYK2_2**	TYR	P
**TYRO3**	TYR	P
**YES1**	TYR	P
**ZAP70**	TYR	P

Supporting information references

108. Brenan, L., Andreev, A., Cohen, O., Pantel, S., Kamburov, A., Cacchiarelli, D., Persky, N. S., Zhu, C., Bagul, M., Goetz, E. M., Burgin, A. B., Garraway, L. A., Getz, G., Mikkelsen, T. S., Piccioni, F., Root, D. E., and Johannessen, C. M. (2016) Phenotypic Characterization of a Comprehensive Set of MAPK1 /ERK2 Missense Mutants. *Cell Rep*. **17**, 1171–1183.
109. Jha, S., Morris, E. J., Hruza, A., Mansueto, M. S., Schroeder, G. K., Arbanas, J., McMasters, D., Restaino, C. R., Dayananth, P., Black, S., Elsen, N. L., Mannarino, A., Cooper, A., Fawell, S., Zawel, L., Jayaraman, L., and Samatar, A. A. (2016) Dissecting Therapeutic Resistance to ERK Inhibition. *Mol Cancer Ther*. **15**, 548–559.
110. Bukhtiyarova, M., Karpusas, M., Northrop, K., Namboodiri, H. V. M., and Springman, E. B. (2007) Mutagenesis of p38α MAP Kinase Establishes Key Roles of Phe169 in Function and Structural Dynamics and Reveals a Novel DFG-OUT State. *Biochemistry*. **46**, 5687–5696.
111. Moretti, S., De Falco, V., Tamburrino, A., Barbi, F., Tavano, M., Avenia, N., Santeusanio, F., Santoro, M., Macchiarulo, A., and Puxeddu, E. (2009) Insights into the molecular function of the inactivating mutations of B-Raf involving the DFG motif. *Biochimica et Biophysica Acta (BBA) - Molecular Cell Research*. **1793**, 1634–1645.
112. Chu, Y., Solski, P. A., Khosravi-Far, R., Der, C. J., and Kelly, K. (1996) The Mitogen-activated Protein Kinase Phosphatases PAC1, MKP-1, and MKP-2 Have Unique Substrate Specificities and Reduced Activity in Vivo toward the ERK2 sevenmaker Mutation. *Journal of Biological Chemistry*. **271**, 6497–6501.
113. Bott, C. M., Thorneycroft, S. G., and Marshall, C. J. (1994) The sevenmaker gain-of-function mutation in p42 MAP kinase leads to enhanced signalling and reduced sensitivity to dual specificity phosphatase action. *FEBS Lett*. **352**, 201–205.
114. Askari, N., Diskin, R., Avitzour, M., Yaakov, G., Livnah, O., and Engelberg, D. (2006) MAP-quest: Could we produce constitutively active variants of MAP kinases? *Mol Cell Endocrinol*. **252**, 231–240.
115. Smorodinsky-Atias, K., Soudah, N., and Engelberg, D. (2020) Mutations That Confer Drug-Resistance, Oncogenicity and Intrinsic Activity on the ERK MAP Kinases—Current State of the Art. *Cells*. **9**, 129.
116. Pan, X., Pei, J., Wang, A., Shuai, W., Feng, L., Bu, F., Zhu, Y., Zhang, L., Wang, G., and Ouyang, L. (2022) Development of small molecule extracellular signal-regulated kinases (ERKs) inhibitors for cancer therapy. *Acta Pharm Sin B*. **12**, 2171–2192.
